# Modeling Pathway Dynamics of the Skeletal Muscle Response to Intravenous Methylprednisolone (MPL) Administration in Rats: Dosing and Tissue Effects

**DOI:** 10.3389/fbioe.2020.00759

**Published:** 2020-07-14

**Authors:** Alison Acevedo, Debra DuBois, Richard R. Almon, William J. Jusko, Ioannis P. Androulakis

**Affiliations:** ^1^Department of Biomedical Engineering, Rutgers University, Piscataway, NJ, United States; ^2^Department of Pharmaceutical Sciences, School of Pharmacy and Pharmaceutical Sciences, State University of New York at Buffalo, Buffalo, NY, United States; ^3^Department of Biological Sciences, State University of New York at Buffalo, Buffalo, NY, United States; ^4^Department of Chemical and Biochemical Engineering, Rutgers University, Piscataway, NJ, United States; ^5^Department of Surgery, Rutgers Robert Wood Johnson Medical School, New Brunswick, NJ, United States

**Keywords:** omics, corticosteroids, pathway analysis, methylprednisolone, dose comparison, tissue comparison

## Abstract

A model-based approach for the assessment of pathway dynamics is explored to characterize metabolic and signaling pathway activity changes characteristic of the dosing-dependent differences in response to methylprednisolone in muscle. To consistently compare dosing-induced changes we extend the principles of pharmacokinetics and pharmacodynamics and introduce a novel representation of pathway-level dynamic models of activity regulation. We hypothesize the emergence of dosing-dependent regulatory interactions is critical to understanding the mechanistic implications of MPL dosing in muscle. Our results indicate that key pathways, including amino acid and lipid metabolism, signal transduction, endocrine regulation, regulation of cellular functions including growth, death, motility, transport, protein degradation, and catabolism are dependent on dosing, exhibiting diverse dynamics depending on whether the drug is administered acutely of continuously. Therefore, the dynamics of drug presentation offer the possibility for the emergence of dosing-dependent models of regulation. Finally, we compared acute and chronic MPL response in muscle with liver. The comparison revealed systematic response differences between the two tissues, notably that muscle appears more prone to adapt to MPL.

## Introduction

Methylprednisolone (MPL) is a synthetic glucocorticoid (GC) widely used to treat a multitude of conditions including arthritis, blood disorders, severe allergic reactions, certain cancers, eye conditions, skin/kidney/intestinal/lung diseases, and immune system disorders. MPL, a typical corticosteroid, manages symptoms such as swelling, pain, and allergic-type reactions by decreasing the immune system’s response (Swartz and Dluhy, 1978; Barnes, 1998). Mechanistic studies of GCs in inflammation have identified two main modes of action: a direct consequence of glucocorticoid/glucocorticoid-receptor complex binding to gene targets, as well as by signaling through receptors in a manner independent of transcription (Schaaf and Cidlowski, 2002; Freishtat et al., 2010). Glucocorticoid effects are pervasive and involve multiple molecular mechanisms. Corticosteroids, including MPL, influence physiology at the regulatory level leading to systemic, multifactorial consequences further complicated by the observed differences in response dynamics to differing dosing regimens of glucocorticoid administration (Almon et al., 2007b; Yao et al., 2008). These changing dynamics are indicative of likely differences in regulatory mechanisms, further revealing that regulatory structures implied by acute administration are not consistent with regulatory structures implied by chronic MPL administration (Hazra et al., 2008; Yao et al., 2008; Nguyen et al., 2010). Acute administration of the drug is generally beneficial by reducing inflammation temporarily. However, chronic administration of corticosteroids, though necessary for chronic conditions, has deteriorative consequences including hyperglycemia, negative nitrogen balance, and fat redistribution leading to complications including diabetes, muscle wasting, osteoporosis (Morand and Leech, 1999; Liu et al., 2017). These consequences are notably observed in muscle where continuous use of corticosteroids leads to muscle atrophy and insulin resistance (Almon et al., 2005a; Schakman et al., 2013; Bodine and Furlow, 2015).

Earlier work has explored *in vivo* high-throughput transcriptomics to capture the tissue and dosing effects of MPL (Sun et al., 1998, 1999; Ramakrishnan et al., 2002a,b; Almon et al., 2005a,b, 2007a, 2008a; Hazra et al., 2007, 2008; Yang et al., 2008, 2009; Yao et al., 2008; Nguyen et al., 2010). We recently proposed a meta-analysis approach to further elaborate our understanding of liver’s complex pharmacogenomic effects following acute and chronic dosing of MPL (Acevedo et al., 2019). The approach applies a pathway-based analysis, mapping transcriptomic data onto tissue- and organism-relevant pathways; characterizes the overall dynamic activity of the pathway; and uses a model-based assessment of activity to infer pathway dynamics. The approach was demonstrated using liver-specific genome-wide pharmacological time-series obtained from comparing alternative dosing regiments.

Given that musculature contributes significantly to adverse glucocorticoid-induced effects, in the present study we employ our established pathway approach to study the acute and chronic MPL dosing effects in gastrocnemius muscle of male adrenalectomized rats, and characterize the dosing-dependent differences in the dynamic response of MPL-responsive pathways. To consistently compare across dosing-induced changes, a model-based approach for the assessment of pathway dynamics is employed extending the principles of pharmacokinetics and pharmacodynamics (PKPD) to characterize pathway activity. We hypothesize the emergence of dosing-dependent regulatory interactions to understand the mechanistic implications of MPL dosing in muscle. Our results indicate that key pathways including amino acid and lipid metabolism, signal transduction, endocrine regulation, regulation of cellular functions including growth, death, motility, transport, protein degradation, and catabolism, are all dependent on dosing. Finally, we compare acute and chronic MPL response across muscle with liver and observe systematic response differences between the two tissues. Notably, we observe that muscle appears more prone to developing tolerance to MPL.

## Materials and Methods

### Animal Model and Experimental Data

The temporal transcriptomic data used for this analysis was collected from extracted gastrocnemius muscle in two temporal large rat studies presented here (Sun et al., 1999; Ramakrishnan et al., 2002a). For the generation of acute MPL response data, 39 adrenalectomized male (ADX) Wistar rats were treated with a bolus dose of 50 mg/kg MPL intravenously (Sun et al., 1999). This dose was established previously for identifying biomarkers for gene-mediated effects of glucocorticoids in liver tissue because of its induction of strong, but not saturating, effects on gene and protein expression and comparability with large doses in human upon scale-up (Boudinot et al., 1986). The animals were sacrificed at 17 timepoints (*n* = 2–4) from 0 to 72 h post dosing and isolated RNA were hybridized with Affymetrix GeneChips Rat Genome U34A containing 8799 probes. Chronic MPL administration response data in muscle tissue was obtained from a longitudinal study in which 40 ADX male Wistar rats were administered 0.3 mg/kg⋅hr of MPL intravenously for 7 days (Nguyen et al., 2010). Animals were sacrificed at 11 time points over this period. Isolated RNA from excised gastrocnemius muscle tissue was hybridized with Affymetrix GeneChips Rat Genome 230A) containing 15,967 probes. Both the acute and chronic datasets have been submitted to GEO (acute: GSE490 and chronic: GSE5101) and we have previously presented analyses of the transcription responses (Sun et al., 1999; Almon et al., 2002, 2005a, 2008a,b; Ramakrishnan et al., 2002a; Yao et al., 2008; Fang et al., 2013; Nguyen et al., 2014).

### Pathway Activity Analysis

**FIGURE 1:**
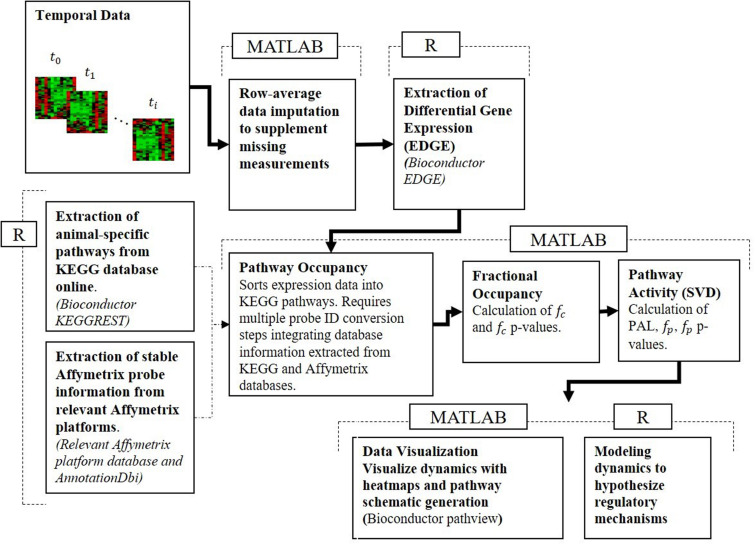
Pathway activity analysis framework starts with the processing of the transcriptional data, maps differentially expressed AffyIDs to gene IDs, assigns those to KEGG pathway gene sets, populates KEGG pathways, assesses fractional occupancy, performs pathway activity to identify significant PALs, and develops dynamic models of regulatory controls of pathway activity.

To reconcile the temporal response of muscle tissue to acute and chronic MPL dosing, the datasets were processed using our pathway activity analysis described in depth in our previous publication analyzing dosing-dependent pathway activity in liver (Acevedo et al., 2019). The approach consists of a series of steps described briefly herein, moved beyond an individual gene-centric analysis, which seeks to characterize muscle response at the level of functional groups – at the level of pathways. For this analysis, pathways are defined as networks of molecular interactions and reactions designed to link genes in the genome to gene products through biochemical action. The steps are succinctly presented in Figure 1.

#### Microarray Data Preprocessing

Active genes are identified using differential expression analysis using the Extraction and Analysis of Gene Expression (EDGE) software (Storey et al., 2018). Differentially expressed profiles are then *z*-scored with respect to the individual profile mean and standard deviation.

#### Mapping Transcriptomic Data to Pathways

Differentially expressed genes are mapped onto pathways, defined as networks of molecular interactions and reactions designed to link genes in the genome to gene products. These pathways express layered and complementary activities, meaning pathways are groups of genes linked mechanistically that effect a signaling or biochemical action. Numerous databases exist defining pathways including the Kyoto Encyclopedia of Genes and Genomes (KEGG) (Aoki and Kanehisa, 2005) and Reactome (Fabregat et al., 2018). Without loss of generality, the present analysis is based on KEGG. As of February 2019, this database contains 326 pathways relevant to rat tissues and used for our analysis. Only pathways relevant to muscle tissue are analyzed resulting in 179 metabolic and signaling pathways for consideration in our analysis (Supplementary Table A). For the gene-to-pathway mapping, Affymetrix probe identifiers within the microarray template and are converted into KEGG IDs in order to be sorted into rat-relevant pathways from KEGG. Affymetrix probe identifiers are translated into their NCBI Entrez IDs and Gene Symbols using the Bioconductor packages for each Affymetrix Platform: Package rae230a.db containing the annotation data for Affymetrix Rat Expression Set 230A used with the chronic data; and Package rgu34a.db containing the annotation data for Affymetrix Rat Genome U34 Array annotation data used with the acute data. Following the gene-to-pathway assignment, the coverage of the KEGG pathways is assessed by evaluating the fractional coverage (*f*_*c*_) of each pathway (Acevedo et al., 2019). This statistic is the fraction of genes within a pathway for which gene profiles are available. To assess the confidence in the fractional coverage, an associated *p*-value (*f*_*c*_ *p*-value) is determined using the 1-tail Fisher’s Exact test such that the total rat genome is the set of unique rat genes in all KEGG’s rat-relevant pathways (Acevedo et al., 2019). Pathways with low fractional occupancy yield inconclusive *p*-values as an artifact of the Fisher’s Exact Test and were eliminated from the analysis.

#### Pathway Activity Analysis

The presence of differentially expressed genes within a pathway does not guarantee that the pathway exhibits a coherent dynamic response (Kallio et al., 2011). In order to assess the emergence of activity patterns within a pathway we capitalize on our earlier work on pathway activity analysis (Ovacik et al., 2010; Euling et al., 2011, 2013) recently expanded in Acevedo et al. (2019). In brief, singular value decomposition (SVD) on the temporal transcriptomic data associated with each pathway, decomposes the overall pathway dynamics into constitutive elements (singular vectors referred to herein as pathway activity levels, PAL) reflecting coherence of expression among the genes of the pathway. The singular value associated with each singular vector expresses the fractional variability (*f*_*p*_) captured by the corresponding PAL. In order to characterize the actual significance of a corresponding PAL, an associated *f*_*p*_ *p*-value is evaluated using bootstrapping of the original gene set (see Supplementary Note) (Acevedo et al., 2019). Finally, all pathways yielding fractional coverage *f*_*c*_ *p*-value ≤0.05 with at least one significant PAL profile *f*_*p*_ *p*-value ≤0.05 are defined as significant. These significance criteria indicate that the pathway is sufficiently represented by the transcriptomic data and that at least one global, non-random, trend has emerged from the pathway. It is important to emphasize that the activity analysis does not make any assumptions as to the nature of the dynamics of the activity across a pathway.

### Evaluating Pathway Activity Dynamics

In order to capture the likely variability of the transcriptomic data bootstrapping is used to generate pathway gene sets likely to exist within the experimental variability. Each bootstrapped gene set is assessed for pathway activity, thus revealing a likely the range of activity a pathway in muscle tissue can produce in response to MPL administration. Briefly, each gene expression profile is bootstrapped assuming a normal distribution about the gene expression profile’s mean. These bootstrapped genes are assembled into pathway gene sets. Thus, (*N* = 1000) bootstrapped pathway gene sets are generated from the original pathway gene set. These bootstrapped sets are decomposed with SVD, significant PAL profiles identified, and their corresponding *f*_*p*_ and *f*_*p*_ *p*-value statistics retained for each significant pathway. All PAL profiles extracted from these bootstrapped gene sets are assumed likely system behavior that would emerge if the rat experiments were repeated. Bootstrapped PAL within a pathway are subsequently clustered for the identification of common activity patterns. The MATLAB^®^ function evalclusters.m is applied to assess optimal cluster number using the gap statistic and applying kmeans clustering (MATLAB, 2018) (see Supplementary Note). The finite set of PAL centroids identified indicate a finite list of activity patterns that emerge from each pathway, induced by MPL. Pathway activity analysis identifies a pathway’s leading intrinsic dynamics as a result of application of its decomposition technique. We seek to compare pathway activities across non-overlapping gene sets and identified from data with different dosing regimens and time horizons. To this end, the dynamics of each dominant PAL is approximated using PKPD-driven models exploring alternative hypotheses for the mechanisms of regulation of a pathway, herein referred to as *pathway pharmacodynamics*.

#### Pharmacokinetics

**FIGURE 2:**
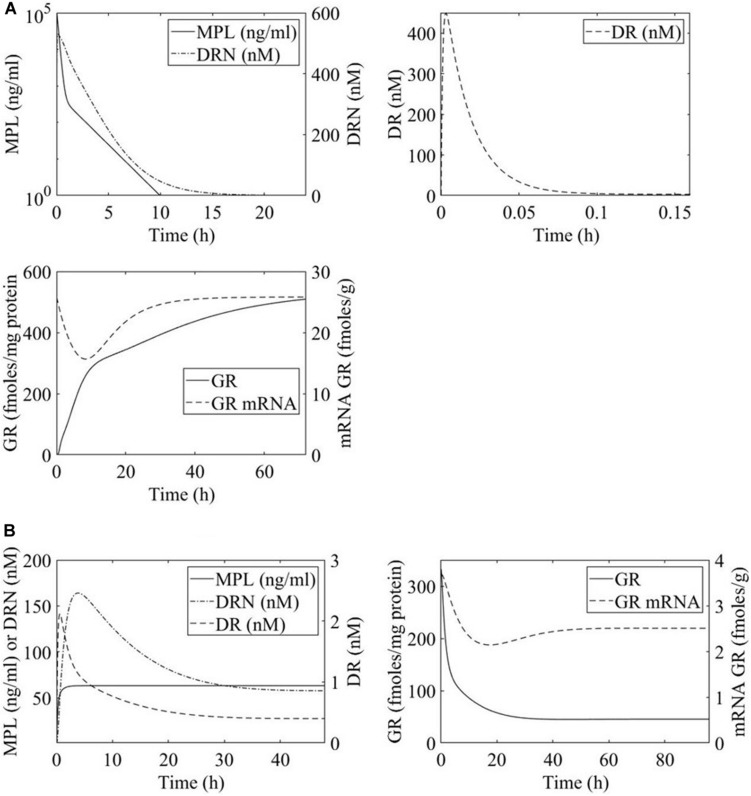
Time profiles of methylprednisolone (MPL) pharmacokinetics and receptor dynamics for **(A)** acute 50 mg/mL bolus MPL dose and **(B)** chronic infusion of 0.3 mg/(kg⋅h) MPL. Methylprednisolone influence over transcription within the liver is dosing dependent and receptor mediated.

The PK of MPL in both regimens was shown to be appropriately described by a two-compartment model, Figure 2, equations 1 and 2 (Ramakrishnan et al., 2002a; Hazra et al., 2008). *A*_*p*_ and *A*_*t*_ denote drug in the plasma and tissue compartments respectively. Term *k*_0_ is the zero-order rate constant for drug input into the plasma, *CL* indicates clearance, *V*_*p*_ indicates plasma volume of distribution, and *k*_12_ and *k*_21_ are the intercompartmental distribution rate constants. In the case of acute MPL administration, *k*_0_ = 0 indicating a bolus injection. Parameter values are adopted from Ramakrishnan et al. and presented in Table 1 (Ramakrishnan et al., 2002a; Hazra et al., 2008).

**TABLE 1:** Pharmacokinetic parameters for acute and chronic MPL administration (Ramakrishnan et al., 2002a).

Parameter	Definition	Acute	Chronic
$k_{0}\,\left(\frac{m\,g}{k\,g}\,h\right)$	Rate of drug infusion into central plasma compartment	0	220
$C\,L\,\left(\frac{L}{h\cdot k\,g}\right)$	Clearance	3.48	5.61
$V_{p}\,\left(\frac{L}{k\,g}\right)$	Central volume of drug distribution	0.73	0.82
$k_{12}\,\left(\frac{1}{h}\right)$	Drug distribution rate constant	0.98	0.32
$k_{21}\,\left(\frac{1}{h}\right)$	Drug distribution rate constant	1.78	0.68

(1) $$\frac{d\,A_{p}}{d\,t}=k_{0}+k_{21}\cdot A_{t}-\left(k_{12}+\frac{C\,L}{V_{p}}\right)\cdot A_{p}$$

(2) $$\frac{d\,A_{t}}{d\,t}=k_{12}\cdot A_{p}-k_{21}\cdot A_{t}$$

#### Receptor Dynamics

MPL action is receptor-mediated as described in equations 3 through 6 and depicted in Figure 2 (Ramakrishnan et al., 2002a; Hazra et al., 2008). Parameter values are adopted from Hazra et al. and presented in Table 2 (Hazra et al., 2008). These parameter values are also used in previous analyses of dosing-dependence in liver (Acevedo et al., 2019). *R*_m_ indicates mRNA of the free cytosolic receptor, *R* indicates the free cytosolic receptor, *DR* indicates the cytosolic drug-receptor complex, and *DRN* indicates the drug-receptor complex in the nucleus (Ramakrishnan et al., 2002a). The concentration at which the synthesis rate of receptor mRNA drops to 50% of its baseline value is indicated by *IC*_50Rm_, parameter *k*_*on*_ denotes a second-order rate constant for drug-receptor binding. Parameters *k*_T_ and *k*_re_ are first-order rates of receptor translocation between the nucleus and the cytosol (*k*_re_ : to the nucleus; *k*_re_ : recycling back to the nucleus) (Ramakrishnan et al., 2002a). The fraction of receptor recycled is indicated by parameter *R*_f_. *C*_*MPL*_ corresponds to the concentration of free receptor in the cytosol and is given by $C_{M\,P\,L}=0.43\,\frac{A_{p}}{V_{p}}$ (Ramakrishnan et al., 2002a; Hazra et al., 2008).

**TABLE 2:** Parameters for receptor-mediated effects of acute and chronic MPL administration (Hazra et al., 2008).

Parameter	Definition	Acute	Chronic
$k_{s\,R\,m}\,\left(\frac{f\,m\,o\,l}{g\cdot h}\right)$	Receptor mRNA synthesis rate constant	3.15	0.45
$k_{d\,R\,m}\,\left(\frac{1}{h}\right)$	Receptor mRNA degradation rate constant	0.122	
$I\,C_{50\,R\,m}\,\left(\frac{n\,m\,o\,l}{L\cdot m\,g_{p\,r\,o\,t\,e\,i\,n}}\right)$	DRN required for 50% inhibition of the synthesis rate of Rm	123.7	
$k_{s\,R}\,\left(\frac{n\,m\,o\,l}{L\cdot m\,g_{p\,r\,o\,t\,e\,i\,n}\cdot f\,m\,o\,l_{R\,m}\cdot g\cdot h}\right)$	Receptor synthesis rate	0.84	3.63
$k_{r\,e}\,\left(\frac{1}{h}\right)$	Loss rate for drug receptor in the nucleus	0.402	
$k_{o\,n}\,\left(\frac{1}{n\,m\,o\,l\cdot h}\right)$	Association rate for receptor-drug binding	0.019	
$k_{d\,R}\,\left(\frac{1}{h}\right)$	Receptor loss/degradation rate	0.0403	
$k_{T}\,\left(\frac{1}{h}\right)$	Translocation of receptor into the nucleus	58.1	
*R*_*f*_	Receptor recycling factor from nucleus to cytosol	0.69	

(3) $$\frac{d\,R\,m}{d\,t}=k_{s\,R\,m}\cdot \left(1-\frac{D\,R\,N}{I\,C_{50\,R\,m}+D\,R\,N}\right)-k_{d\,R\,m}\cdot R\,m$$

(4) $$\frac{d\,R}{d\,t}=k_{s\,R}\cdot R\,m+R_{f}\cdot k_{r\,e}\cdot D\,R\,N-k_{o\,n}\cdot C_{M\,P\,L}\cdot R-k_{d\,R}\cdot R$$

(5) $$\frac{d\,D\,R}{d\,t}=k_{o\,n}\cdot C_{M\,P\,L}\cdot R-k_{T}\cdot D\,R$$

(6) $$\frac{d\,D\,R\,N}{d\,t}=k_{T}\cdot D\,R-k_{r\,e}\cdot D\,R\,N$$

#### Pathway Pharmacodynamics

Pharmacogenomic models have been extensively used to model complex transcriptional dynamics (Almon et al., 2002; Ramakrishnan et al., 2002b; Jin et al., 2003; Yao et al., 2008; Ayyar et al., 2018), whereas we recently extended the concept to describe complex “pathways” pharmacodynamics (Acevedo et al., 2019). We hypothesize that transcriptional events are induced by the regulatory action of an MPL-receptor complex (DRN) binding to a GRE element in the nucleus. In order to capture more complex behaviors, such as tolerance and rebound, it has been hypothesized that the receptor complex can likely induce intermediate biosignal (BS) inducing complex responses (Sharma et al., 1998; Sharma and Jusko, 1998). By decomposing the pathway dynamics to its constitutive PALs, we aim to characterize the dosing-dependent activity in muscle, by hypothesizing that each PAL can be represented by an appropriate dynamic model. We thus compare PAL dynamics across dosing, and tissues, in the space of pathway pharmacodynamic models. PAL profiles were captured by our “receptor-mediated” or “biosignal-mediated” model types as previously discussed in the context of liver (Acevedo et al., 2019) and developed as an extension of the concepts presented in Hazra et al. (2008) and Yao et al. (2008).

**FIGURE 3:**
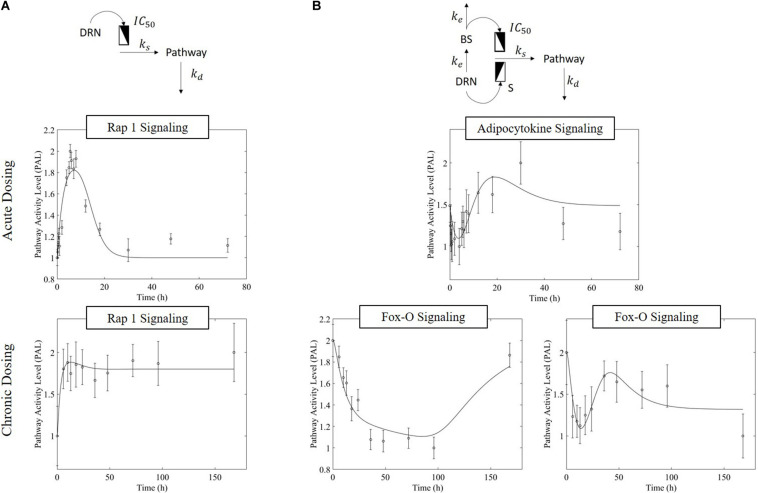
Regulatory mechanism schematics for the **(A)** receptor-mediated regulation of PAL and **(B)** biosignal-mediated regulation of PAL. Methylprednisolone regulates transcription via binding to glucocorticoid receptors within the cytosol, transporting into the nucleus, and binding to a GRE element – thus initiating targeted transcription. (*Receptor-mediated dynamics*) Since the activity is driven by the levels of the active signal DRN, and this follows the PK of MPL, acute dosing the response dynamics should exhibit a major event with a subsequent return to pre-administration levels (exemplified by Acute Rap 1 Signaling), as the PK of MPL prescribes. Under chronic dosing, since the levels of DRN stabilize at a new steady state, the DRN effect should persist for as long as the levels of DRN are constant (exemplified by acute Rap 1 signaling response). (*Receptor-mediated and biosignal-mediated dynamics*) If the drug induces the release of a secondary biosignal (BS) this will induce a delayed secondary effect which, if competing with the effect of DRN, would lead to a “rebound”-type of response. As MPL clears, and both DRN and BS deplete, the system returns to pre-MPL level (exemplified by Adipocytokine signaling). Chronic administration can generate two distinct types of dynamics responses (the presence of the secondary biosignal BS can induce a secondary event, competing with that of DRN). BS can exercise its impact either in a delayed manner leading to a “tolerance-like” behavior (exemplified by Fox-O Signaling response, **A**) or through an incomplete rebound effect with the system relaxing eventually at a steady state other than the pre-MPL one (exemplified by Fox-O Signaling response, **B**). For each pathway, the significant bootstrapped PAL are clustered such that common activity patterns group together. PAL profiles plotted herein are plots of the centroids of these clusters (represented by the data points), fitted with models (represented by the fitted continuous profile). The error bars about the PAL central data points are defined by the standard deviation of all bootstrapped PAL that are captured within the cluster.

The receptor-mediated model (Figure 3A, equation 7) indicates a mode of pathway regulation which assumes a saturable induction of the pathway activity driven primarily by the active MPL-receptor complex (*k*_s_ indicates the activation rate of pathway activity; *IC*_50PAL_ indicates the concentration of DRN responsible for 50% inhibition of the pathway activity activation rate; and *k*_*d*_ indicates the deactivation rate of pathway activity). This model captures the pathway activity response to intravenous MPL administration for both acute and chronic dosing, reflecting transient or persistent response types depending on dosing. Receptor-mediated response is expected to taper-off under acute administration since the dynamics of *PAL* should follow the dynamics of *DRN*. Under chronic dosing, and as *DRN* accumulates the influence should persist (Figure 3).

(7) $$\frac{d\,P\,A\,L}{d\,t}=k_{s}\,\left(1\pm \frac{D\,R\,N}{I\,C_{50\,P\,A\,L}+D\,R\,N}\right)-k_{d}\cdot P\,A\,L$$

MPL regulation can be also mediated via an intermediate biosignal whose synthesis is directly related to DRN (Yao et al., 2008), equations 8 and 9 (*k*_e_ indicates the translation rate of BS; *S* is the stimulation constant for pathway activity due to DRN; *IC*_50PAL_ indicates the BS responsible for 50% inhibition of pathway activity activation rate; and γ indicates the factor of amplification of the influence of BS on the activation of pathway activity). Biosignal-mediated responses could either lead to “rebound” like behavior under acute dosing or, under chronic dosing, could lead to “tolerance”-like response or “rebound”-like eventually reaching a new steady state (Figure 3)

(8) $$\frac{d\,B\,S}{d\,t}=k_{e}\,\left(D\,R\,N-B\,S\right)$$

(9) $$\frac{d\,P\,A\,L}{d\,t}=k_{s}\,\left(1\pm S\cdot D\,R\,N\right)\,\left(1\mp \frac{B\,S^{\gamma }}{I\,C_{50\,P\,A\,L}^{\gamma }+B\,S^{\gamma }}\right)-k_{d}\cdot P\,A\,L$$

As previously discussed in Acevedo et al. (2019), the parameter estimation was performed using MATLAB’s optimization toolkit in a series of optimization stages. In all stages, we sought to minimize the residual sum of squares between the model prediction and the cluster centroid profile. In the first stage, it is assumed that the system is non-linear and neither continuous nor differentiable for the entire parameter solution space. Therefore, as a rapid preliminary global search for a minimum, a stochastic direct method (simulated annealing) with bound constraints is employed. The result of this global search technique is taken as the initial parameter values for the second optimization stage using a direct pattern search method. In the final stage, a gradient-based method is used to probe this more limited space as the final optimization step. This stage uses the sequential quadratic programming as implemented through MATLAB’s fmincon. The model which results from this optimization process is visually inspected.

## Results

**FIGURE 4:**
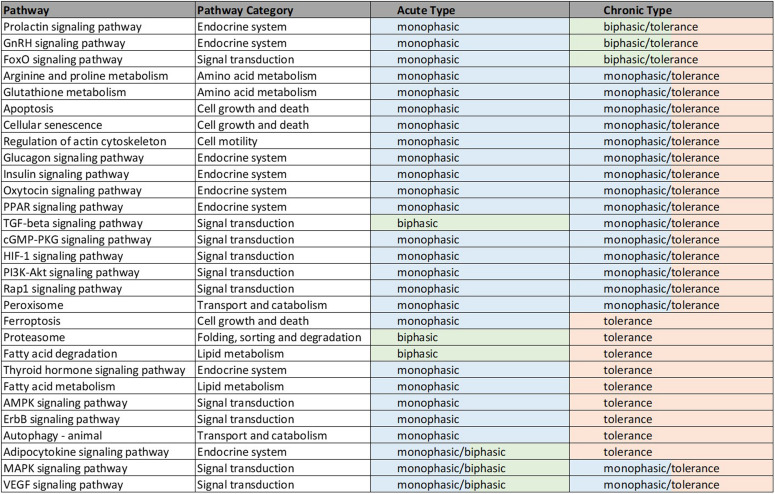
Illustrated table capturing the summarized responses of each of the 29 significant pathways common to the acute and chronic muscle analyses. The consistent response types are indicative of likely common effects of MPL across metabolic and signaling pathways. The details of these dynamics are included in Supplementary Table D.

Of the 179 pathways determined to be rat- and muscle- relevant, fractional coverage analysis yielded 51 represented pathways in the acute dataset and 61 in the chronic dataset. Pathway activity analysis examined these pathways to determine whether significant PALs emerged from each pathway. Pathways which yielded at least one significant PAL (*f*_*p*_ *p*-value ≤0.05) were considered active. For the acute dosing, 49 pathways emerged as significant while all 61 pathways emerged as significant for the chronic dosing (Significant pathways counts are listed by subgroup in Table 3, organized by subgroup. They are also listed in long form name with KEGG identification information in Supplementary Tables STB, STC), chronic dosing appears to engage relatively more of the amino acid and carbohydrate metabolism function of the tissue, whereas acute dosing appears to drive lipid metabolism and induce relatively more activity in endocrine and signaling functions. A subset of 29 pathways were identified as significant in both acute and chronic dosing (Table 3 and Figure 4) comprising a collection of metabolic, signaling and endocrine functions. The dynamic profiles of all 29 pathways, in acute and chronic dosing, are presented in the Supplementary Figures and the pathways are listed in the Supplementary Tables. When compared against the list of significant pathways in liver, we identified a subset of pathways that were also significant in liver in response to acute and chronic MPL administration, detailed further in the “Discussion” section. Broadly, chronic dosing appears to engage relatively more amino acid and carbohydrate metabolism pathways compared to acute dosing.

**TABLE 3:** Counts of pathways that emerged as significant in response to acute and chronic MPL dosing in muscle.

Pathway subgroup	Pathway group	Significant acute response pathways	Significant chronic response pathways
Cell growth and death	Cellular processes	5	3
Cell motility	Cellular processes	1	1
Transport and catabolism	Cellular processes	3	6
Signal transduction	Environmental information processing	17	15
Signaling molecules and interaction	Environmental information processing	1	0
Folding, sorting and degradation	Genetic information processing	1	4
Transcription	Genetic information processing	0	1
Translation	Genetic information processing	0	1
Amino acid metabolism	Metabolism	2	6
Carbohydrate metabolism	Metabolism	0	9
Energy metabolism	Metabolism	0	2
Lipid metabolism	Metabolism	4	2
Metabolism of cofactors and vitamins	Metabolism	0	1
Metabolism of other amino acids	Metabolism	1	1
Nucleotide metabolism	Metabolism	1	0
Xenobiotics biodegradation and metabolism	Metabolism	1	0
Endocrine system	Organismal systems	12	8
Environmental adaptation	Organismal systems	0	1
	**Total**	**49**	**61**

*See Supplementary Tables for complete list of significant pathways and all calculated metrics.*

**FIGURE 5:**
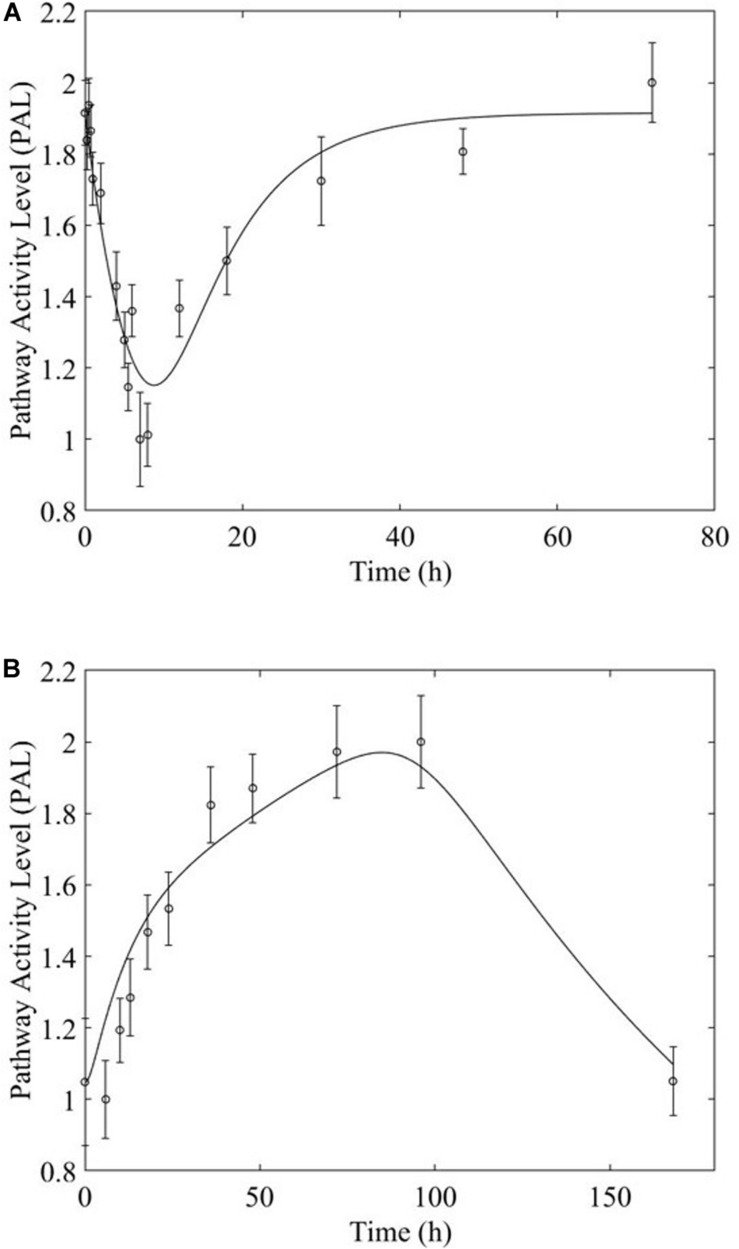
AMPK signaling pathway response to **(A)** acute and **(B)** chronic MPL administration.

**FIGURE 6:**
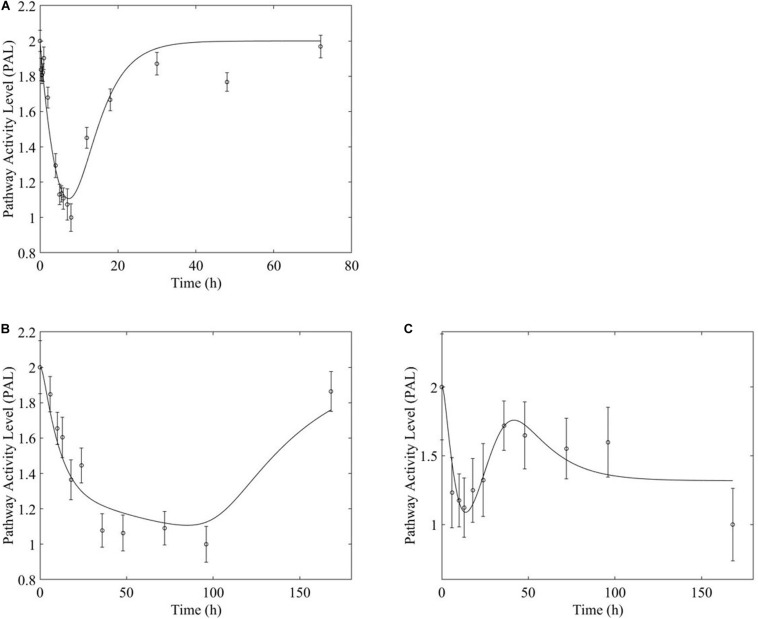
Fox-O signaling pathway response to (**A**, top) acute and (**B,C**, bottom) chronic MPL administration.

**FIGURE 7:**
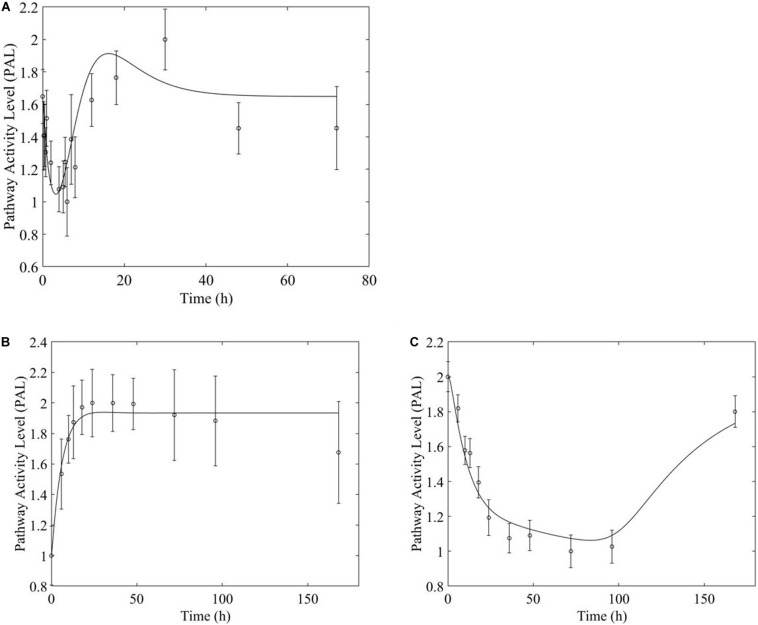
PPAR signaling pathway response to (**A,** top) acute and (**B,C**, bottom) chronic MPL administration.

Overwhelmingly, muscle responds in a relatively direct way to acute dosing. Except for a handful of pathways, muscle response to acute dosing is receptor mediated. A transient response is observed in pathway profiles shortly after MPL dosing. This initial response is transient, and the pathway eventually resolves to the pre-administration levels. Characteristic examples of this response type are observed in AMPK, Fox-O, and PPAR signaling pathways (Figures 5A, 6A, 7A). These results are generally consistent with the observed PK of MPL since the drug clears within about 10 h (Figure 2).

**FIGURE 8:**
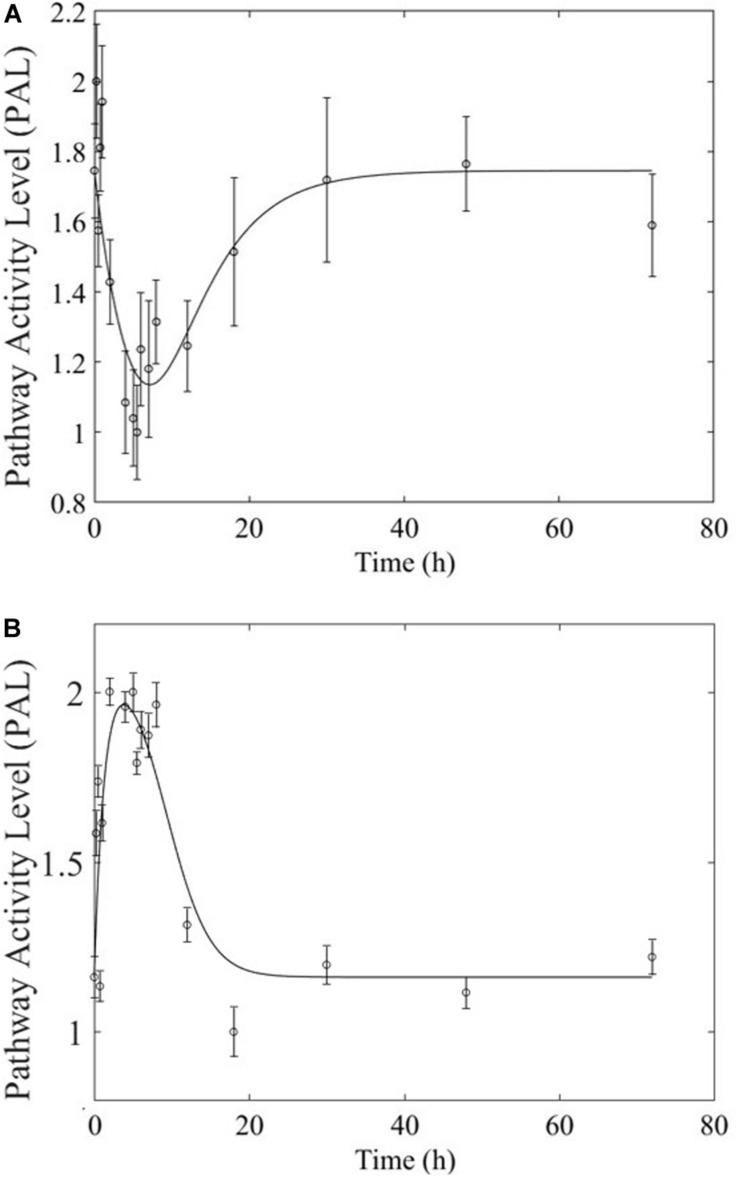
Acute MPL administration in muscle **(A)** and liver **(B)**. Receptor mediated dynamics dominate acute administration in both tissues, as illustrated here for the peroxisome pathway.

**FIGURE 9:**
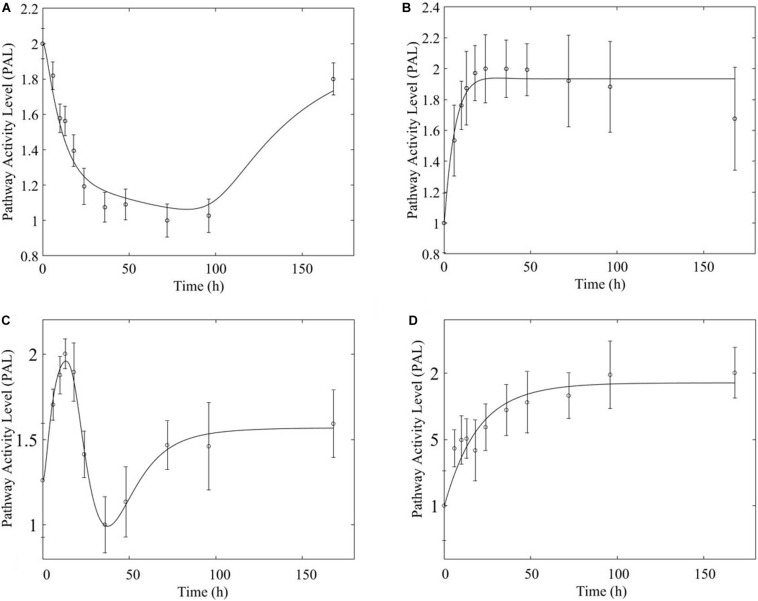
Comparison of response to chronic MPL administration in muscle (**A,B**, top) and liver (**C,D**, bottom) for PPAR. Both tissues exhibit more complex behavior exhibiting a combination of biosignal-mediated (**A,C**, left) and receptor-mediated effects (**B,D,** right).

**FIGURE 10:**
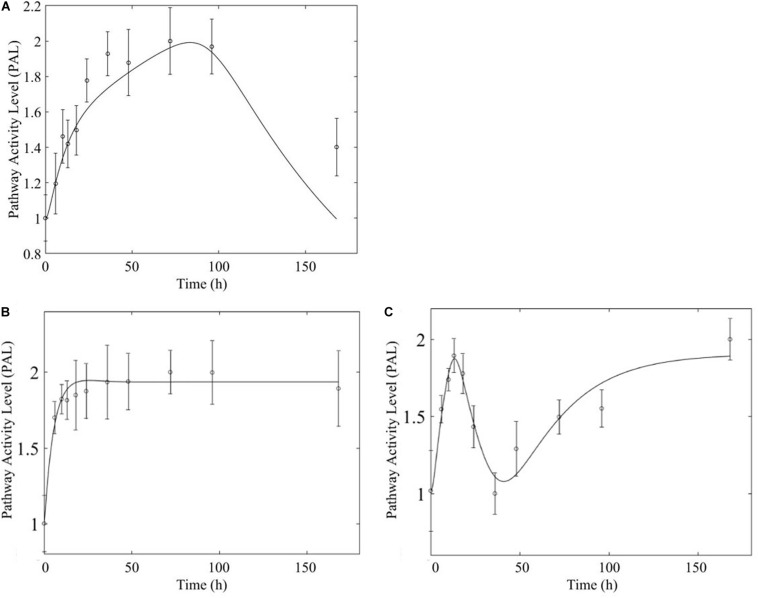
Comparison of response to chronic MPL administration in muscle (**A,** top) and liver (**B,C**, bottom) of fatty acid degradation. The pathway analysis reveals a biosignal-mediated tolerance in muscle.

**FIGURE 11:**
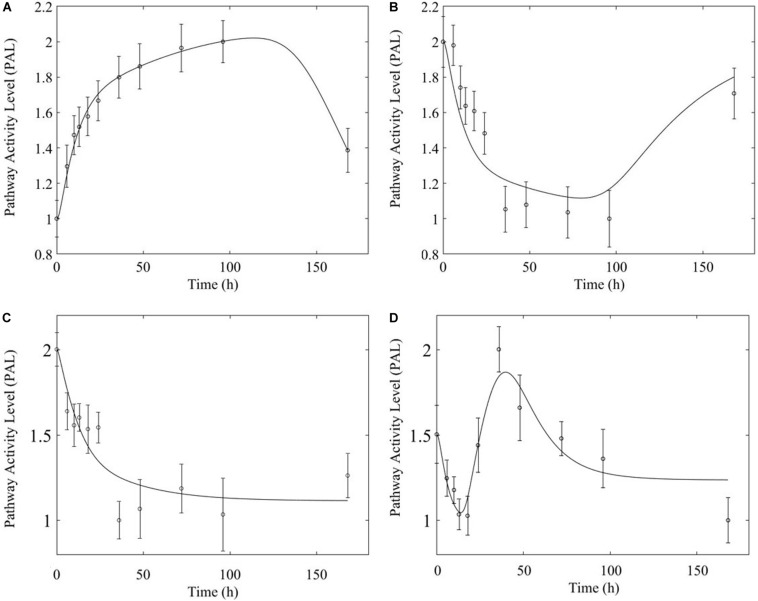
Comparison of response to chronic MPL administration for arginine biosynthesis (left: **A,C**) and valine, leucine, isoleucine degradation (right: **B,D**). The top panels depict response in muscle while the bottom panels depict response in liver.

Based on our earlier studies which determined that acute and chronic MPL exposure impact differentially liver (Acevedo et al., 2019), we hypothesize the drug to induce dosing-dependent and tissue-specific effects, as earlier transcriptomic analyses indicate (Ballard et al., 1974; Sun et al., 1999; Yao et al., 2008; Nguyen et al., 2014). When comparing liver and muscle effects, we determine that acute dosing elicits comparable dynamics in liver and muscle, as exemplified in Figure 8 for the peroxisome signaling pathway for which the dominant regulatory structure is receptor-mediated. However, chronic dosing appears to drive: (1) substantially different response dynamics in the two tissues, as exemplified with chronic administration of MPL impact to the PPAR signaling pathway (Figure 9); (2) simple(r) muscle dynamics but more complex liver dynamics, illustrated with fatty acid degradation pathway (Figure 10); and (3) leading dynamics manifested via tissue-specific regulation, or time scales (Figure 11).

Chronic MPL administration elicits complex behaviors in muscle. Despite a continuous infusion of the drug, the muscle-specific pathway dynamic responses relax over the 170 h experimental time course to either a new steady state or, via some mechanism of tolerance, returns to the pre-exposure condition. Examples of this tolerance behavior are described Supplementary Table D and depicted in greater detail in the Supplementary Figures. The new steady state is achieved via multiple mechanism options: (1) by the equilibrating roles of DRN and BS (AMPK, Figure 5B); (2) by a combination of the previous mechanism and a “rebound” effect leading to part of the pathway reaching a different steady state (Fox-O, Figures 6B,C); (3) by a combination of equilibration of the DRN and BS forces and the persistent regulatory driver toward a new steady state (PPAR, Figures 7B,C). One of the most striking observations of this analysis is that muscle appears to better “tolerate” chronic MPL exposure with a variety of mechanisms, enabling the tissue to recover to pre-exposure features of pathway dynamics.

## Discussion

MPL is a widely used anti-inflammatory and immune-suppressing drug. Like most drugs, its use can exhibit both deleterious and beneficial effects. Specifically with respect to muscle, chronic use has been associated with muscle atrophy and insulin resistance (Schakman et al., 2013; Bodine and Furlow, 2015). Despite its continued use, the mechanistic details are not entirely characterized. Multiple dosing studies aim at probing the system differentially and as such hold promise in terms deciphering some of those complexities. Reconciling data from multiple studies that explore the influence of MPL is not straightforward, as data can potentially be collected using different experimental platforms, on different time scales, within different tissues, and across different dosing regimens (Ghosh et al., 2003; Ramasamy et al., 2008; Tseng et al., 2012).

Pathway activity analysis was explored to identify pathways that are significantly active in response to MPL. We view a (metabolic or signaling) pathway as a high-dimensional system whose decomposition identifies intrinsic trends. Using singular value decomposition (SVD), a pathway’s dynamic is decomposed into constituent singular values and singular vectors. The singular vectors are linear combinations of the longitudinal transcriptional expression over time, thus capture trends in the activity of the pathway, defined as Pathway Activity Level (PAL). The fraction of variability of each PAL profile is calculated from the singular values and is defined as the fraction of pathway activity (*f*_*p*_). Alternatively, a PAL profile can be thought of as the expression of a metagene over time, where the metagene is a representation of common trends in gene expression within a pathway. The emergence of multiple significant PALs indicates a codominance of activity patterns, and complex regulatory structures, within the pathway. To characterize these activity dynamics consistently, we extended the PD concept to describe the dynamics of a signaling or metabolic pathway’s corticosteroid impact in muscle tissue. Models that describe receptor and biosignal-mediated regulation by MPL were fitted to PAL profiles. This step served to hypothesize likely modes of pathway regulation and is essential to enable comparison of drug response across experimental platforms, animals, tissues, dosing regimens, or time horizons. Overall, we observed strikingly consistent response profiles across pathways and within the same dosing regimen. We further observed that chronic administration yielded more complex pathway dynamics for most pathways, than did acute administration.

We first analyze the MPL response in each dosing regimen independently in the context of functional groups, i.e., pathways, and characterize this in the space of regulatory models. The authors would like to acknowledge that the acute platform is smaller than the microarray platform used to generate the chronic dataset. This detail of our data is consistent with our previous analysis in liver (Acevedo et al., 2019). The significant chronic pathways generally yield higher fractional coverage than the acute counterparts, and this is likely due to the difference in platforms. As a result of this, it may be true that the differences in gene content bias the pathway dynamics. However, the purpose of the fc p-value is to identify whether a pathway is sufficiently represented in our data and thus indicates whether we can trust a pathway to be represented in our solution set. It is this analysis step and statistic which enables us to retain as much data as possible for both studies in order to assess pathway dynamics without reducing one platform or the other to only a subset of genes common between the platforms. Further, because both acute and chronic studies capture the influence of MPL within muscle tissue, a consistent set of pathways is anticipated to emerge, and is observed (Table 4), when comparing these data, further discussed later in this section.

**TABLE 4:** Significant pathways common to acute and chronic MPL administration in muscle.

Pathway	rno ID	Genes in KEGG pathway	Acute				Chronic			
			Unique genes in pathway from dataset	Fractional pathway coverage (fc)	Total fraction of pathway activity (total fp)	Acute MPL response	Unique genes in pathway from dataset	Fractional pathway coverage (fc)	Total fraction of pathway activity (total fp)	Chronic MPL response
**Amino acid metabolism**										
Arginine and proline metabolism	rno00330	52	9	17%	42%	Receptor-mediated	17	33%	56%	Receptor-mediated/tolerance
Glutathione metabolism	rno00480	69	10	14%	69%	Receptor-mediated	22	32%	72%	Receptor-mediated/tolerance
**Cell growth and death**										
Apoptosis	rno04210	141	23	16%	61%	Receptor-mediated	38	27%	55%	Receptor-mediated/tolerance
Cellular senescence	rno04218	189	24	13%	57%	Receptor-mediated	44	23%	69%	Receptor-mediated/tolerance
Ferroptosis	rno04216	41	9	22%	49%	Receptor-mediated	15	37%	72%	Tolerance
**Cell motility**										
Regulation of actin cytoskeleton	rno04810	223	21	9%	47%	Receptor-mediated	48	22%	71%	Receptor-mediated/tolerance
**Endocrine system**										
Adipocytokine signaling pathway	rno04920	74	14	19%	57%	Receptor-mediated/biosignal-mediated	20	27%	55%	Tolerance
Glucagon signaling pathway	rno04922	103	11	11%	65%	Receptor-mediated	32	31%	58%	Receptor-mediated/tolerance
GnRH signaling pathway	rno04912	94	12	13%	60%	Receptor-mediated	29	31%	76%	Biosignal-mediated/tolerance
Insulin signaling pathway	rno04910	138	18	13%	50%	Receptor-mediated	43	31%	73%	Receptor-mediated/tolerance
Oxytocin signaling pathway	rno04921	157	21	13%	58%	Receptor-mediated	40	25%	57%	Receptor-mediated/tolerance
PPAR signaling pathway	rno03320	82	15	18%	61%	Receptor-mediated	21	26%	61%	Receptor-mediated/tolerance
Prolactin signaling pathway	rno04917	74	13	18%	61%	Receptor-mediated	18	24%	48%	Biosignal-mediated/tolerance
Thyroid hormone signaling pathway	rno04919	118	21	18%	41%	Receptor-mediated	30	25%	55%	Tolerance
**Folding, sorting and degradation**										
Proteasome	rno03050	48	14	29%	62%	Biosignal-mediated	30	63%	87%	Tolerance
**Lipid metabolism**										
Fatty acid degradation	rno00071	47	11	23%	44%	Biosignal-mediated	18	38%	70%	Tolerance
Fatty acid metabolism	rno01212	59	12	20%	59%	Receptor-mediated	19	32%	68%	Tolerance
**Signal transduction**										
AMPK signaling pathway	rno04152	127	16	13%	50%	Receptor-mediated	36	28%	58%	Tolerance
cGMP-PKG signaling pathway	rno04022	172	19	11%	54%	Receptor-mediated	38	22%	53%	Receptor-mediated/tolerance
ErbB signaling pathway	rno04012	88	11	13%	70%	Receptor-mediated	23	26%	61%	Tolerance
FoxO signaling pathway	rno04068	134	16	12%	61%	Receptor-mediated	43	32%	68%	Biosignal-mediated/tolerance
HIF-1 signaling pathway	rno04066	107	16	15%	55%	Receptor-mediated	35	33%	67%	Receptor-mediated/tolerance
MAPK signaling pathway	rno04010	300	39	13%	59%	Receptor-mediated/biosignal-mediated	68	23%	81%	Receptor-mediated/tolerance
PI3K-Akt signaling pathway	rno04151	349	45	13%	51%	Receptor-mediated	73	21%	56%	Receptor-mediated/tolerance
Rap1 signaling pathway	rno04015	213	28	13%	54%	Receptor-mediated	44	21%	67%	Receptor-mediated/tolerance
TGF-beta signaling pathway	rno04350	93	11	12%	47%	Biosignal-mediated	23	25%	54%	Receptor-mediated/tolerance
VEGF signaling pathway	rno04370	58	14	24%	57%	Receptor-mediated/biosignal-mediated	20	34%	83%	Receptor-mediated/tolerance
**Transport and catabolism**										
Autophagy - animal	rno04140	134	16	12%	70%	Receptor-mediated	45	34%	59%	Tolerance
Peroxisome	rno04146	88	11	13%	47%	Receptor-mediated	24	27%	77%	receptor-mediated/tolerance

*The pathways exhibit statistically significant fractional coverage (*f*_*c*_*p*-value ≤ 0.05) and significant pathway activity (*f*_*p*_*p*-value ≤ 0.05) in both the acute and chronic dosing. Detailed descriptions of the dynamics observed in all significant pathways are included in Supplementary Table D.*

Forty nine pathways (see Supplementary Table B) were identified as active in the acute set. Interestingly, the dominant regulatory structure is receptor-mediated to acute MPL administration in muscle tissue with a consistent response of an initial peak in activity due to DRN action between 5 and 15 h followed by a return to baseline between 20 and 40 h. This response is consistent with the nature of the acute dosing–an MPL half-life of 0.33 h in ADX rats with total drug clearance observed after about 4.6 h (Hazra et al., 2007). Pathway families represented in this subset include: amino acid metabolism (Arginine and proline metabolism, Glutathione metabolism), pathways related to cell motility, cell growth and death, cellular events such folding, sorting, and degradation of genetic material and proteins, transport, and catabolism (Regulation of actin cytoskeleton, Apoptosis, Cellular senescence, Ferroptosis, Proteasome, Autophagy, Peroxisome), endocrine regulation (Signaling pathways for Glucagon, GnRH, Insulin, Oxytocin, Prolactin, PPAR, and Thyroid hormone), signal transduction (Signaling pathways for TGF-beta, AMPK, cGMP-PKG, ErbB, Fox-O, HIF-1, PI3K-Akt, and Rap1), and lipid metabolism (Fatty acid degradation and Fatty acid metabolism).

The chronic administration explored the Affymetrix microarray platform 230A and yielded 61 pathways as significantly active (Supplementary Table C). As in the acute response analysis, response to chronic administration yielded consistency in profile activity events across pathways. However, pathways varied in their complexity of response to chronic administration by exhibiting receptor and/or biosignal-mediated dynamics, often leading to tolerance, defined herein as a return to baseline despite continuous drug infusion. A tolerance profile is characterized by the receptor-mediated effects of *DRN* and the biosignal-mediated effects of *BS* regulating the activity of the pathway in opposite directions. The AMPK signaling pathway, Thyroid hormone signaling pathway, Autophagy, ErbB signaling pathway, Ferroptosis, and Fatty acid metabolism pathways yield strictly tolerance (biosignal-mediated) response to chronic MPL. The HIF-1 signaling pathway, Regulation of actin cytoskeleton, Oxytocin signaling pathway, PI3K-Akt signaling pathway, Apoptosis, Cellular senescence, Peroxisome, Insulin signaling pathway, Rap1 signaling pathway, Glucagon signaling pathway, cGMP-PKG signaling pathway, Arginine and Proline metabolism, Glutathione metabolism, PPAR signaling pathways exhibit both receptor, as well as biosignal-mediated responses to chronic MPL administration.

Chronic dosing appears to, disproportionately, impact metabolic processes, as indicated in Table 3. While the fraction of signaling pathways, whose activity is impacted by acute or chronic dosing is high for both, chronic dosing appears to engage amino acid and carbohydrate metabolism more actively. One of the most intriguing findings of the study is that the chronic MPL administration does not induce persistent effects on all pathways. This behavior is exemplified by the AMPK signaling pathway (Figure 5), an energy metabolism regulator responsible for inhibiting energy-consuming pathways (anabolic functions) and activating ATP-generating catabolic pathways. Activation of this pathway is unsurprising because it is previously observed that corticosteroid treatment causes mitochondrial dysfunction in muscle cells, which induces a state of ATP deprivation and subsequent activation of AMPK signaling to counteract this, ultimately leading to muscle atrophy (Liu et al., 2015). What is surprising is that in response to chronic MPL, the AMPK pathway yields a biosignal-mediated response. This is indicative of the development of tolerance to MPL because despite continuous administration of MPL over the course of the experiment, the pathway returns to baseline – at least for the duration of the experiment.

However, chronic MPL administration has the potential of yielding more complex behaviors. The Fox-O signaling pathway consists of a series of transcription factors that regulate multiple events within the cell including “apoptosis, cell-cycle control, glucose metabolism, oxidative stress resistance, and longevity (KEGG, 2019a).” Fox-O transcription factors including Foxo1 and Foxo3a are upregulated in response to the corticosteroid dexamethasone and are key regulators of gene expression leading to muscle atrophy (Waddell et al., 2008; Zhao et al., 2009; Schakman et al., 2013). In response to chronic MPL administration, the Fox-O pathway (Figure 6A) yields two formats of biosignal-mediated responses revealing an increased complexity across dosing studies, as well as an internal complexity to the pathway; subgroups of genes within this pathway respond differently to the same chronic dosing regimen. Part of the dominant pathway activity (Figure 6B) indicates the development of tolerance to MPL, marked by the observation that the system returns to baseline despite continued administration of MPL. An additional pathway activity exhibits a biphasic response, eventually settling to a new steady state reflective of persistent MPL effects (Figure 6C). A different combination of the biosignal-mediated tolerance response and persistent receptor-mediated pathway activation is exemplified by the PPAR signaling pathway (Figure 7). This pathway assists in regulating lipid metabolism in liver and skeletal muscle (Burri et al., 2010; KEGG, 2019b) and is implicated in muscle atrophy in response to corticosteroid dexamethasone treatment via the mechanism of PPAR upregulation of Fox-O transcription factor expression in muscle (Castillero et al., 2013). Like Fox-O, PPAR develops two modes of response to chronic administration.

### Liver and Muscle Response to MPL Administration

#### Acute Dosing

The most striking characteristic of muscle, compared to liver, pathway dynamics in response to acute MPL administration, is that muscle was found to be driven primarily by receptor mediated regulation, as opposed to liver which appears to reflect a balance between receptor and biosignal-mediated regulation (Acevedo et al., 2019). However, when the dynamics are driven by receptor binding, the timescale of the response appears to be comparable between the two tissues (Figure 8), likely due to the nature of MPL administration (*intravenous*).

#### Chronic Dosing

In comparing the response of chronic MPL dosing between liver and muscle, the most striking observation is that the tolerance response observed as a major constituent of muscle tissue was rarely observed in the liver (Acevedo et al., 2019). This suggests that in response to chronic MPL administration, muscle tissue can make functional adjustments to restore pre-administration levels, whereas liver is less likely to adjust and settles to a new set point, in most cases. However, intriguing responses do emerge. Figure 9 depicts PPAR signaling dynamics, where a combination of receptor and biosignal regulation, and tolerance is observed, albeit through different mechanisms. In muscle, this biosignal-mediated response manifests as the tolerance-like behavior, whereas in liver a biphasic response emerges, as denoted by the two characteristic peaks in opposite directions corresponding to an initial receptor-mediated event primarily driven by DRN and subsequent rebound-like, secondary, action due to BS. However, both tissues appear to share a, common, second component of the pathway activity leading to a receptor-mediated displacement to a new steady state as MPL, and by extension *DRN*, equilibrates to its new steady state (Figure 9, right panels). Fatty acid degradation, depicted in Figure 10, is another characteristic example of tissue-specific regulation. In muscle (Figure 10, top) the dynamic response of the pathway under conditions of chronic exposure to MPL indicate a combination of receptor- and biosignal-regulation with a tolerance-like behavior. However, the acute response indicates a strictly receptor-mediated response (bottom left) in conjunction with a biosignal-mediated response (right). Furthermore, liver appears to adapt to a long-term response more gracefully, despite continuous presence of the drug. Particularly interesting are pathways that exhibit a single dominant dynamic in each tissue, which manifests in different ways. Two characteristic examples are the arginine biosynthesis and the valine, leucine, isoleucine degradation pathways (Figure 11). Arginine biosynthesis (Figure 11, left panels) reveals a biosignal mediated response in muscle (top) given a sharp increase in activity early on followed by a sharp decline at later times, whereas in liver (bottom) points to a receptor-mediated impact of MPL on the activity of the pathway. The valine, leucine, isoleucine degradation pathway (Figure 11, right panels), critical for protein metabolism, reported in both tissues (Nair et al., 1992; Holeček, 2002; Campos-Ferraz et al., 2013), points to yet another interesting tissue-specificity: most likely both tissues respond via a combination of receptor- and biosignal-mediated regulation. However, muscle (top) exhibits a more protracted response, whereas liver (right) indicates a much faster, rebound-like, dynamic.

Tissue-specificity is a recognized yet underutilized resource particularly in drug discovery (Yang et al., 2018; Yao et al., 2018; Ryaboshapkina and Hammar, 2019). The lack of a detailed understanding of the underlying gene regulation is clearly a major roadblock (Sonawane et al., 2017). The problem is easily stated: even though all tissues carry common genes not all genes perform the same functions or respond the same way. Our earlier studies have explored the co-expression – co-regulation premise to describe regulatory similarities and differences induced by MPL chronic and acute dosing in liver (Nguyen et al., 2010) and muscle (Nguyen et al., 2014). Interestingly, an emerging hypothesis posits that is that tissue-specific regulation is driven by regulatory paths (connections between target genes and transcription factors) rather than activation of tissue-specific transcription factors (Sonawane et al., 2017). Our results extend this concept to also account for dosing, i.e., the dynamics of the external signal presentation in the tissue. Even when considering genes at their functional rather than individual level, it appears as if distinct regulatory modes emerge. The interesting extension is that targeting a single gene may only provide part of the story. If the aim is to either modify or re-establish the functional characteristics of the tissue, the emphasis needs to shift from genes to functional groupings (metabolic and signaling pathways and/or drug modes of action). The proposed work is, we believe, a step in this direction.

## Data Availability Statement

The datasets for this study can be found in the GEO data base https://www.ncbi.nlm.nih.gov/geo/ using identifies for the acute (GSE490 and chronic (GSE5101) administration respectively.

## Author Contributions

AA developed the computational framework, conducted the studies, analyzed the results, co-wrote, and edited the manuscript. DD, RA, and WJ provided the experimental data, contributed to the analysis, and reviewed the manuscript. IA conceived the studies, contributed to the analysis, co-wrote, and edited the manuscript.

## Conflict of Interest

The authors declare that the research was conducted in the absence of any commercial or financial relationships that could be construed as a potential conflict of interest.
