# Supplementary material for: Modeling Pathway Dynamics of the Skeletal Muscle Response to Intravenous Methylprednisolone (MPL) Administration in Rats: Dosing and Tissue Effects
Source: Front Bioeng Biotechnol. 2020 Jul 14;8:759. doi: 10.3389/fbioe.2020.00759 (PMC7371857; doi:10.3389/fbioe.2020.00759)
Supplement: Supplementary file 1 [file Presentation_1.PPTX]

## Slide 1
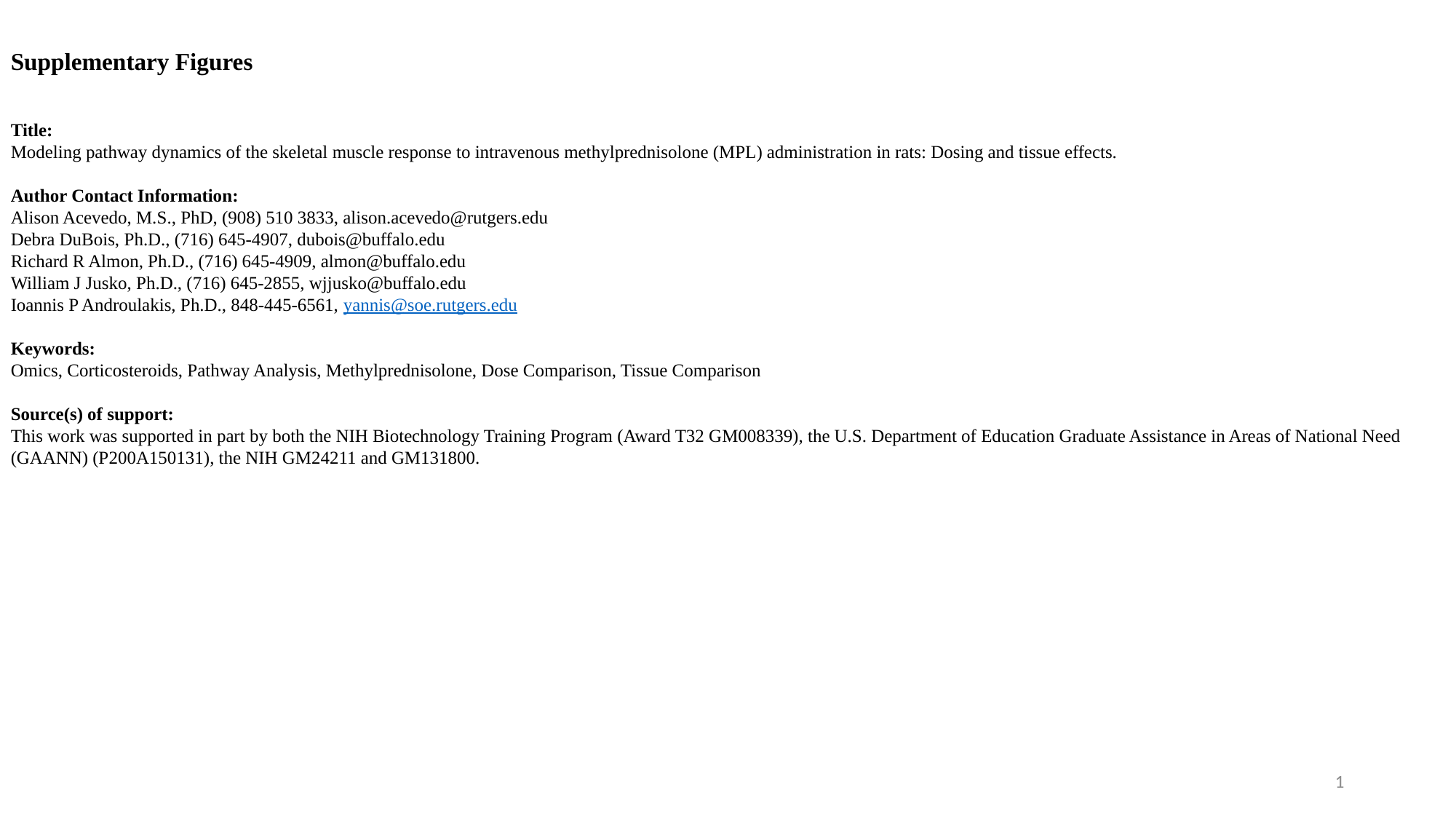

Supplementary Figures
Title:
Modeling pathway dynamics of the skeletal muscle response to intravenous methylprednisolone (MPL) administration in rats: Dosing and tissue effects.
Author Contact Information:
Alison Acevedo, M.S., PhD, (908) 510 3833, alison.acevedo@rutgers.edu
Debra DuBois, Ph.D., (716) 645-4907, dubois@buffalo.edu
Richard R Almon, Ph.D., (716) 645-4909, almon@buffalo.edu
William J Jusko, Ph.D., (716) 645-2855, wjjusko@buffalo.edu
Ioannis P Androulakis, Ph.D., 848-445-6561, yannis@soe.rutgers.edu
Keywords:
Omics, Corticosteroids, Pathway Analysis, Methylprednisolone, Dose Comparison, Tissue Comparison
Source(s) of support:
This work was supported in part by both the NIH Biotechnology Training Program (Award T32 GM008339), the U.S. Department of Education Graduate Assistance in Areas of National Need (GAANN) (P200A150131), the NIH GM24211 and GM131800.
1

## Slide 2
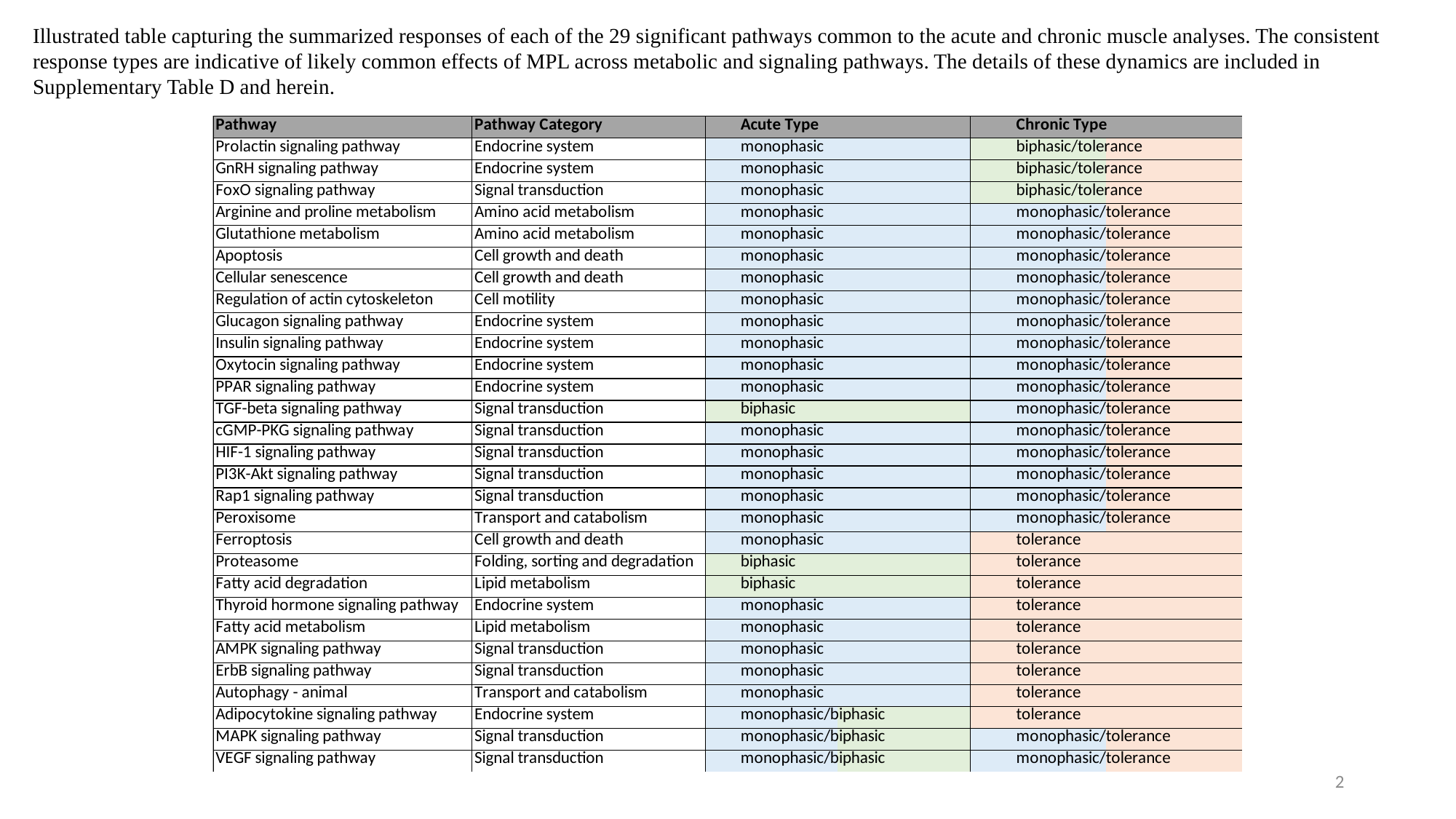

Illustrated table capturing the summarized responses of each of the 29 significant pathways common to the acute and chronic muscle analyses. The consistent response types are indicative of likely common effects of MPL across metabolic and signaling pathways. The details of these dynamics are included in Supplementary Table D and herein.
2

## Slide 3
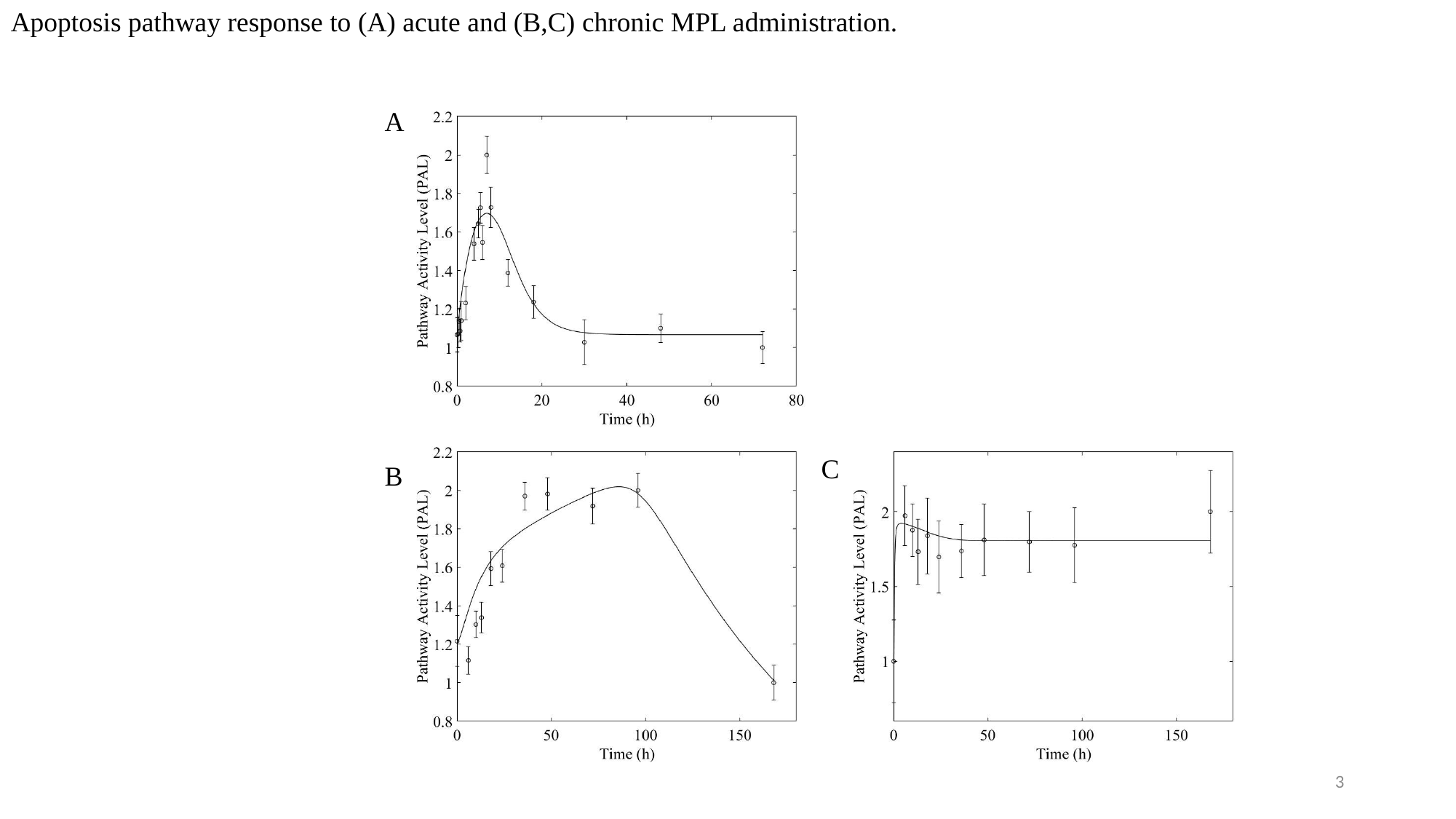

Apoptosis pathway response to (A) acute and (B,C) chronic MPL administration.
A
B
C
3

## Slide 4
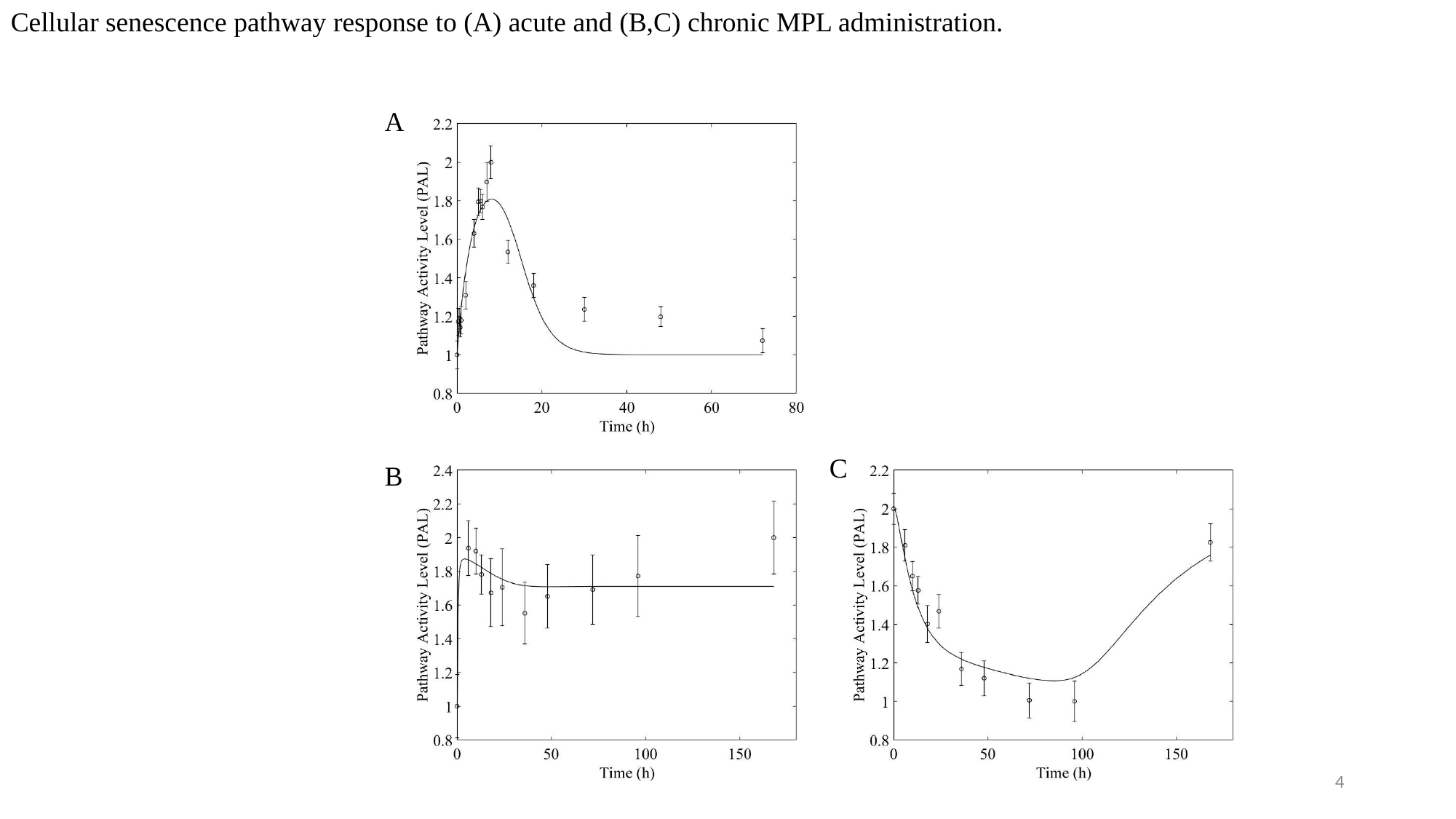

Cellular senescence pathway response to (A) acute and (B,C) chronic MPL administration.
A
B
C
4

## Slide 5
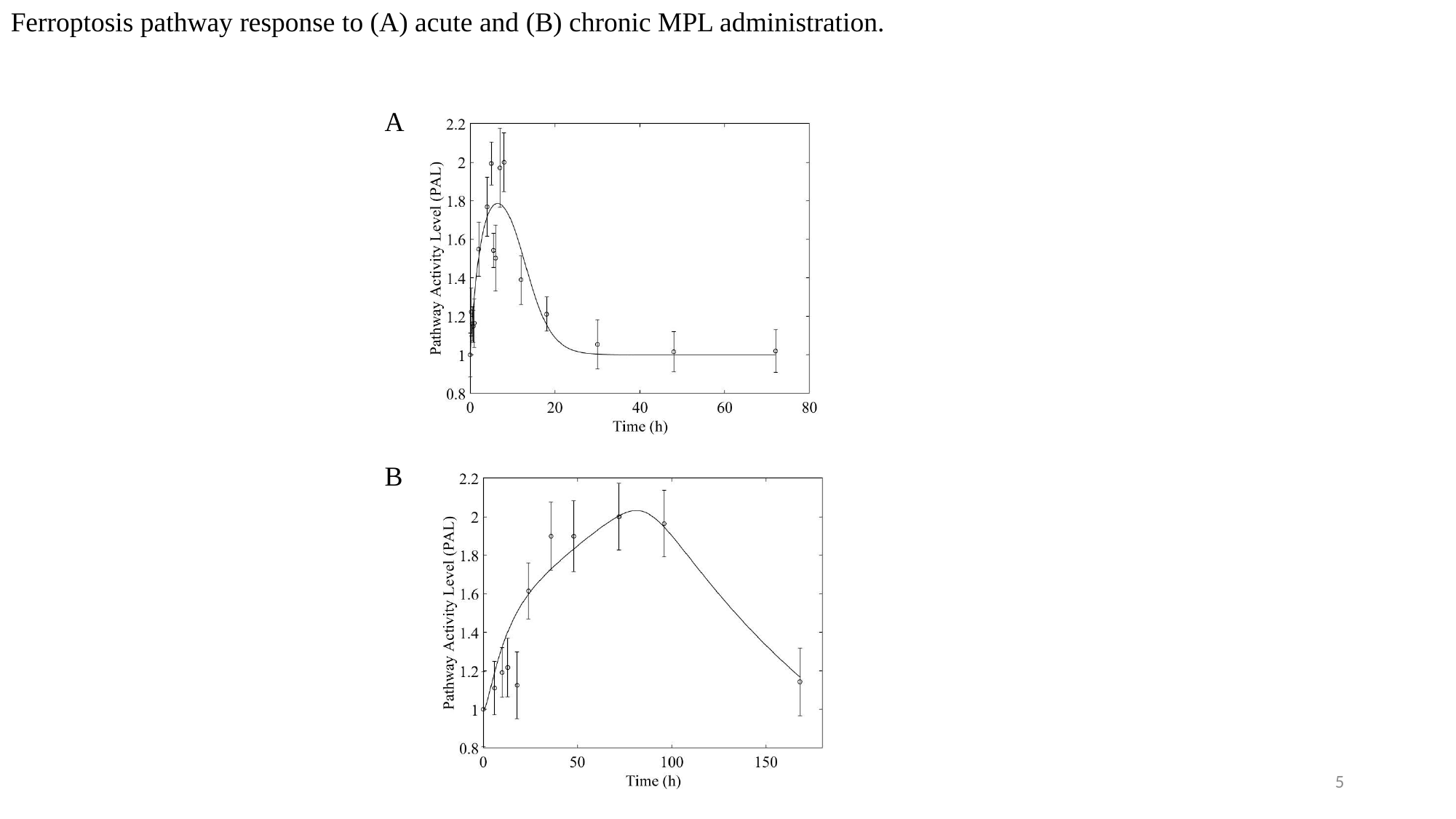

Ferroptosis pathway response to (A) acute and (B) chronic MPL administration.
A
B
5

## Slide 6
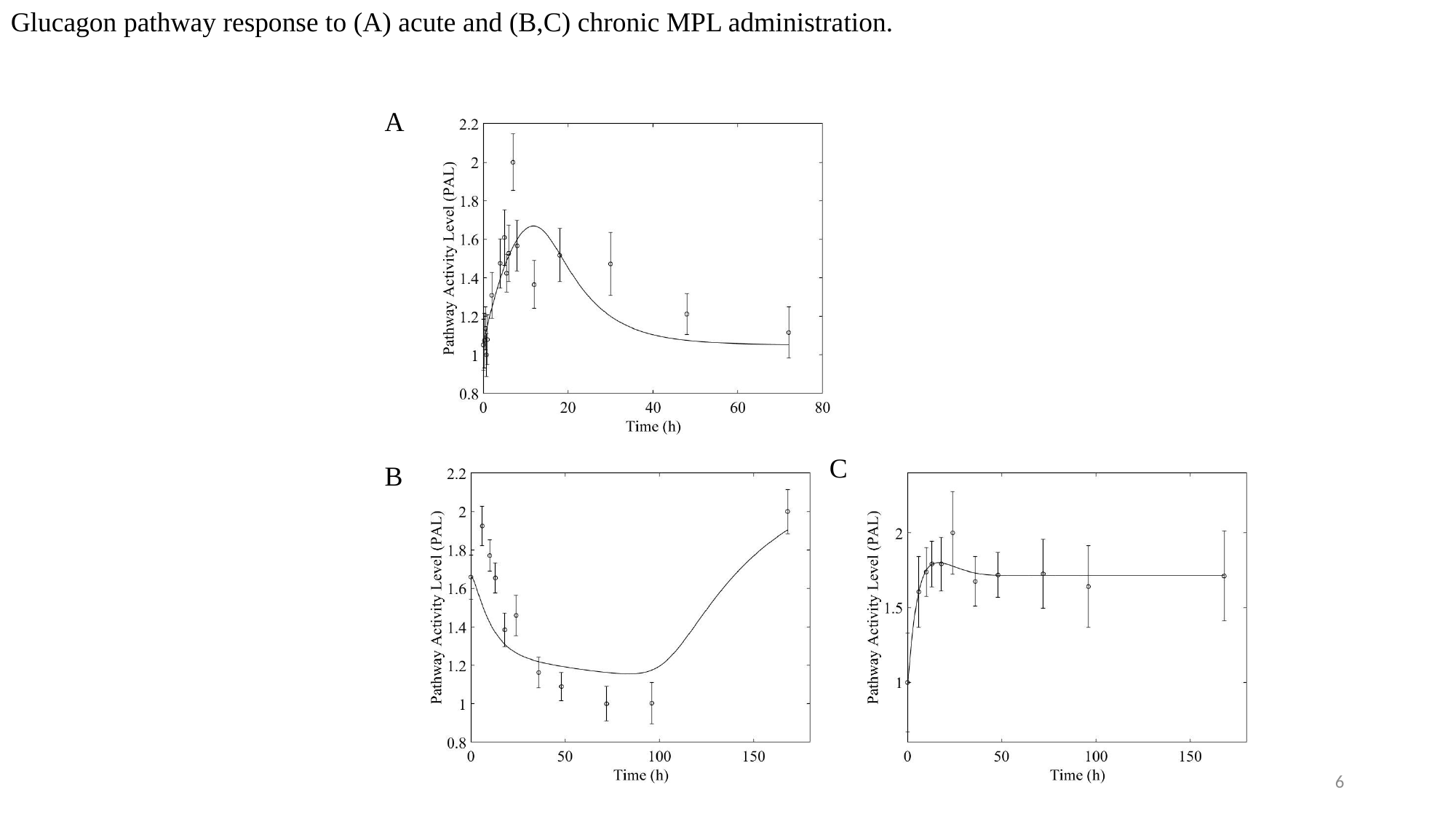

Glucagon pathway response to (A) acute and (B,C) chronic MPL administration.
A
B
C
6

## Slide 7
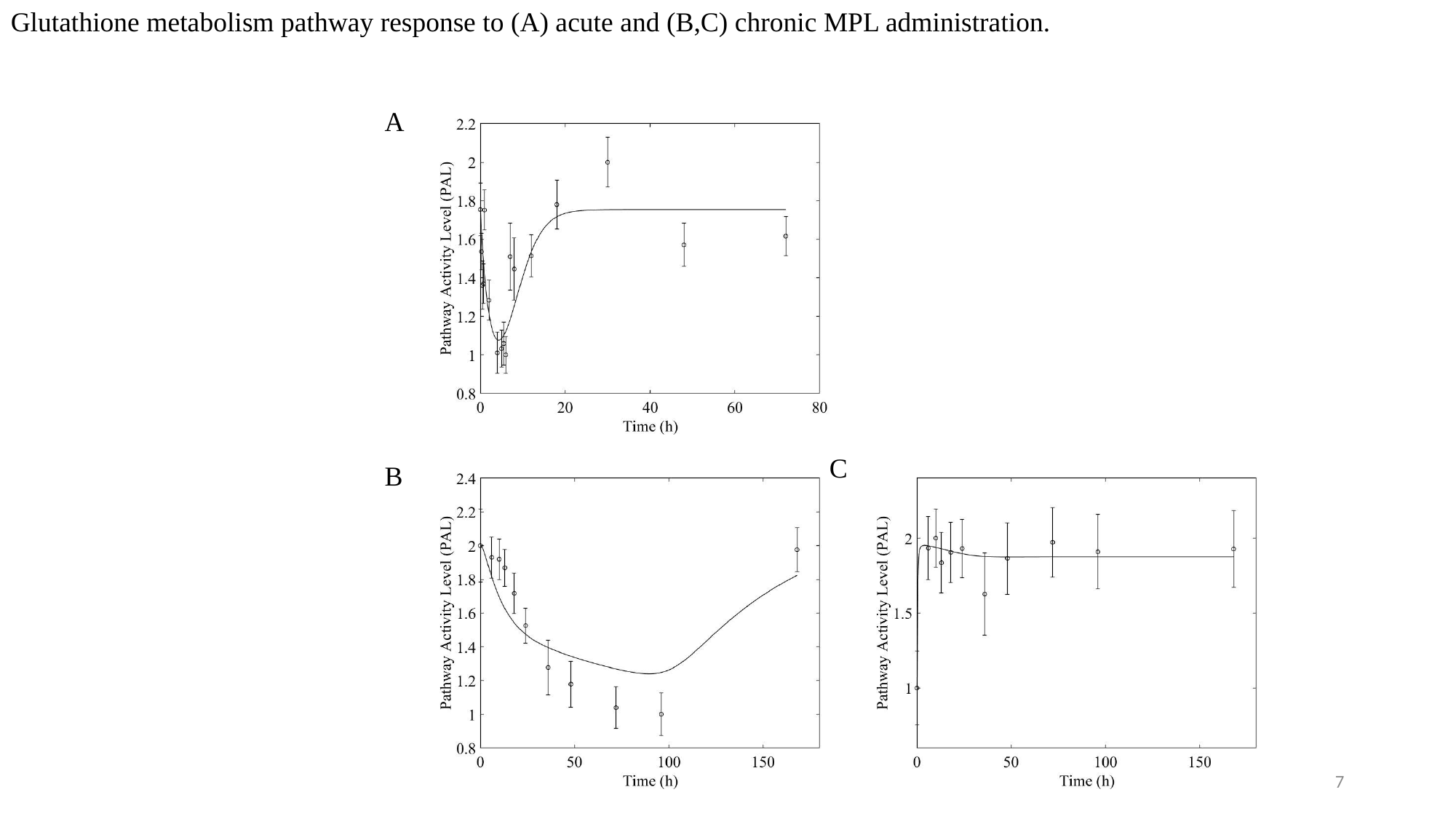

Glutathione metabolism pathway response to (A) acute and (B,C) chronic MPL administration.
A
B
C
7

## Slide 8
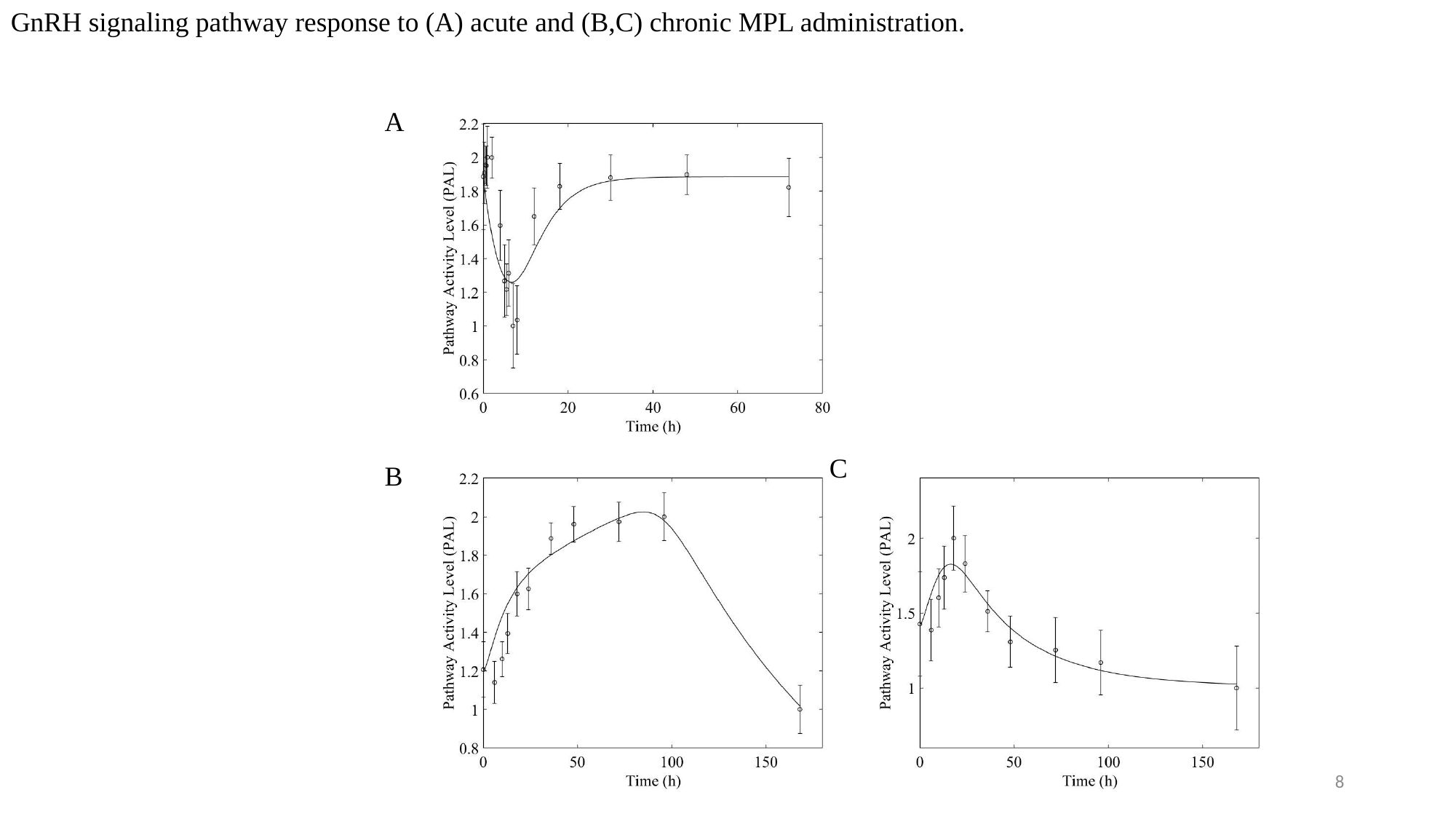

GnRH signaling pathway response to (A) acute and (B,C) chronic MPL administration.
A
B
C
8

## Slide 9
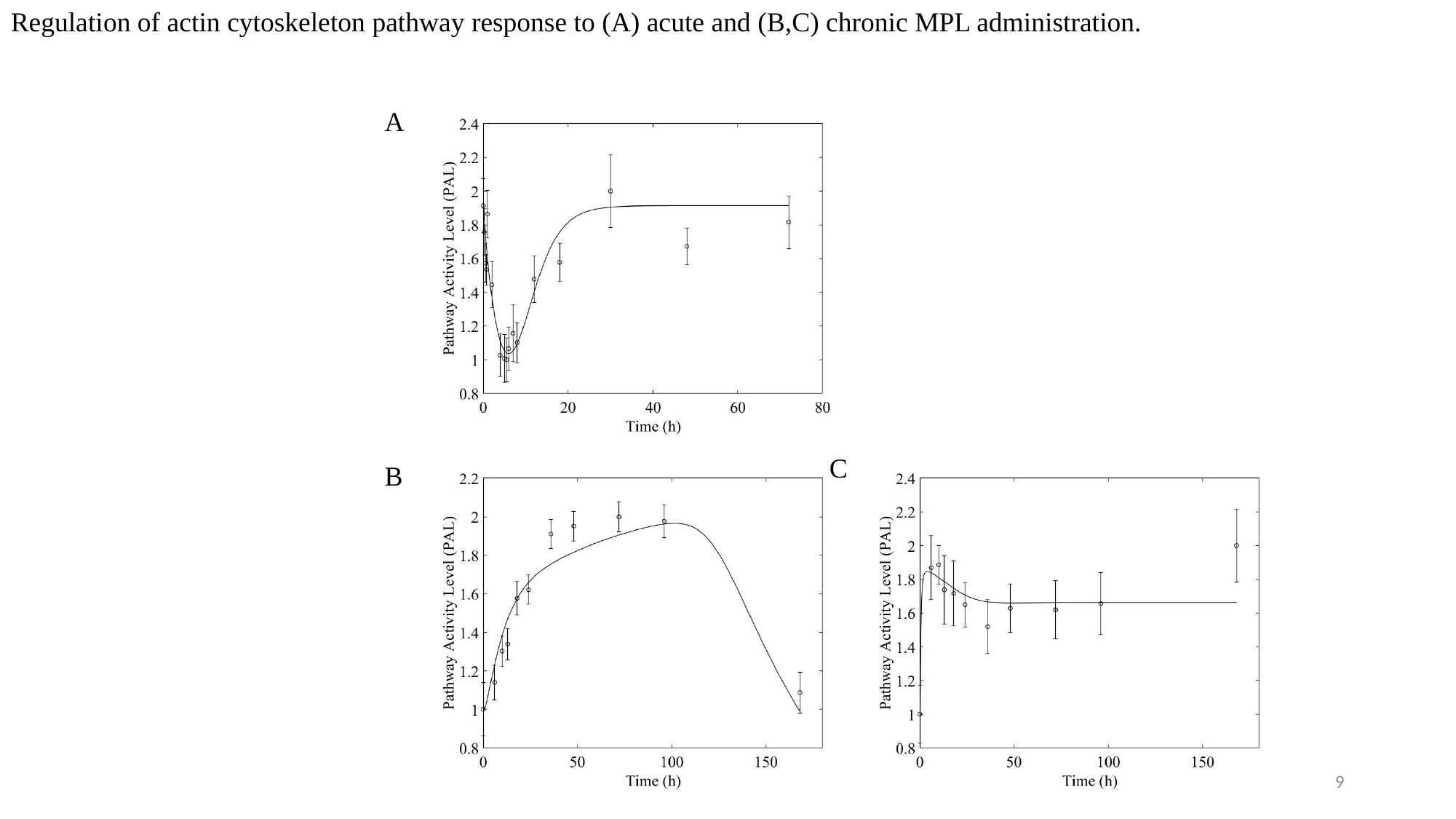

Regulation of actin cytoskeleton pathway response to (A) acute and (B,C) chronic MPL administration.
A
B
C
9

## Slide 10
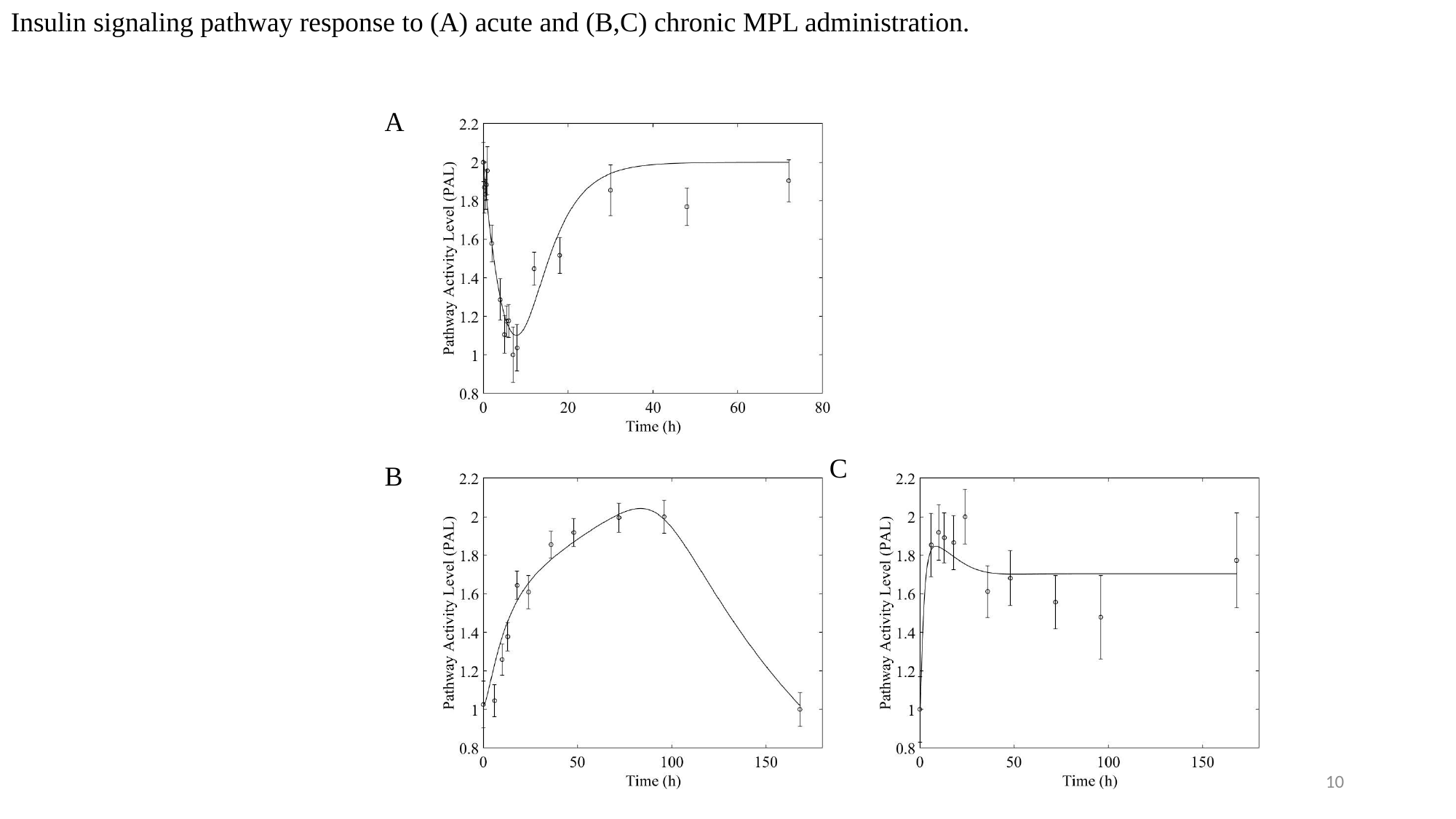

Insulin signaling pathway response to (A) acute and (B,C) chronic MPL administration.
A
B
C
10

## Slide 11
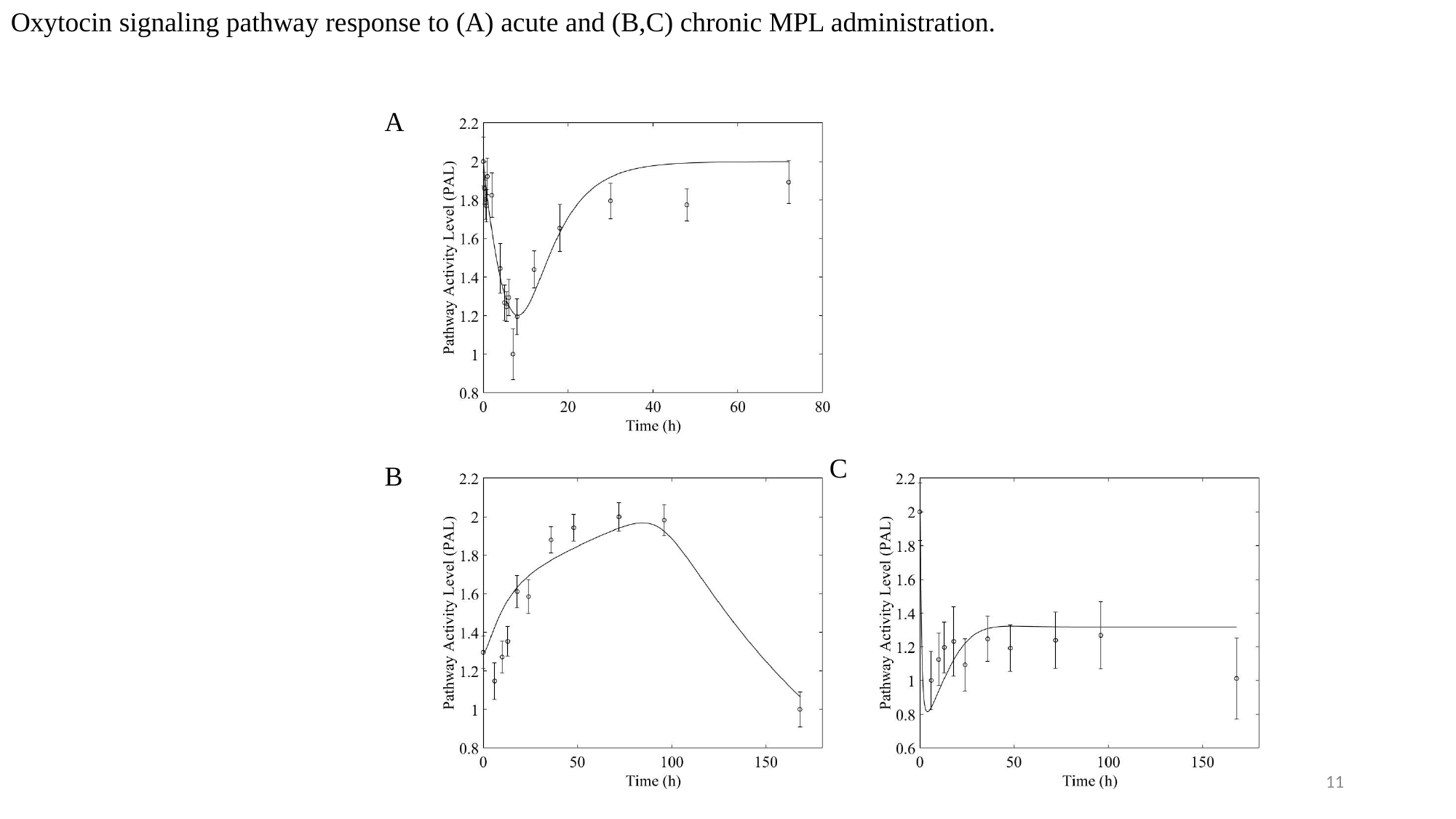

Oxytocin signaling pathway response to (A) acute and (B,C) chronic MPL administration.
A
B
C
11

## Slide 12
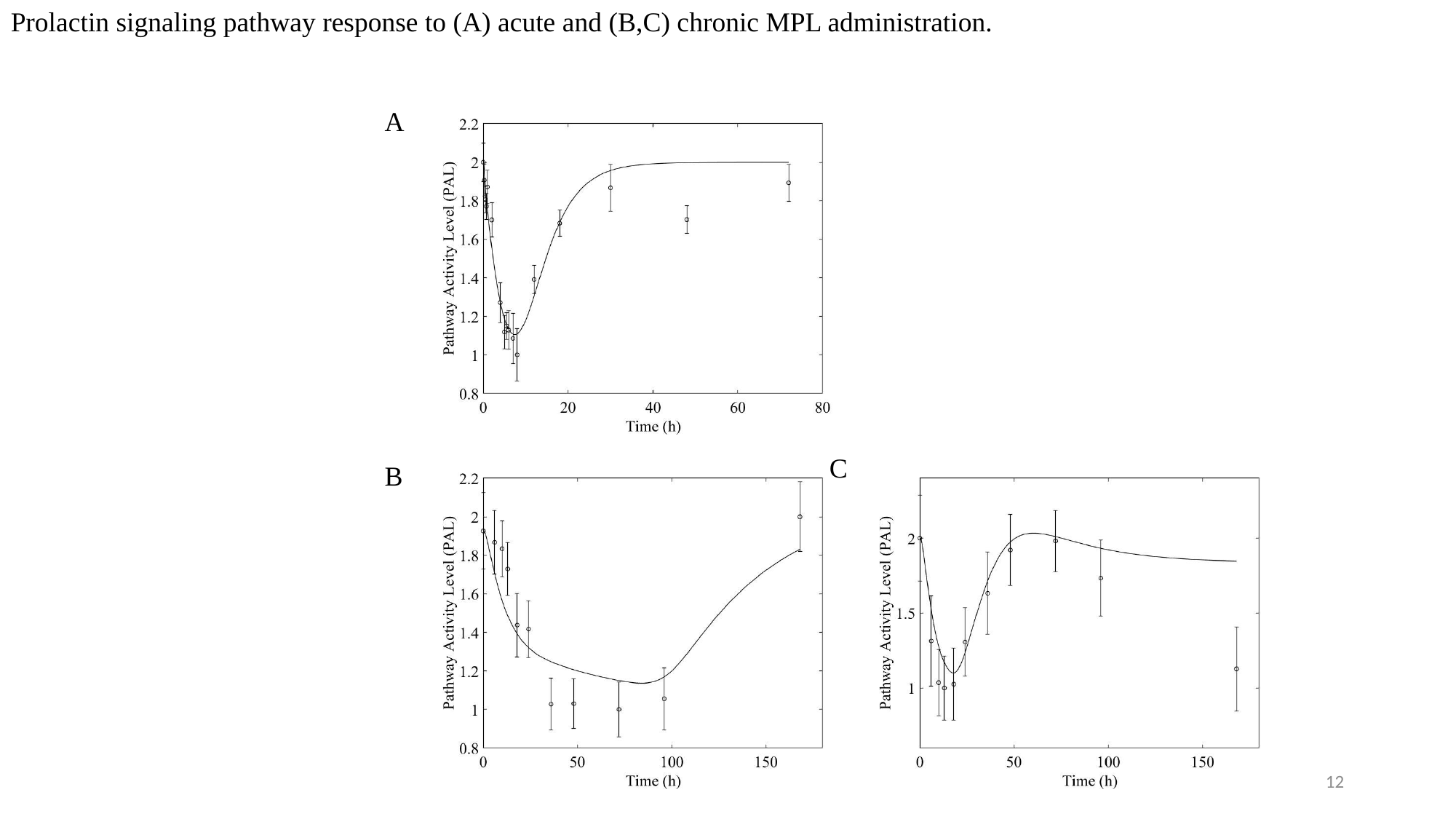

Prolactin signaling pathway response to (A) acute and (B,C) chronic MPL administration.
A
B
C
12

## Slide 13
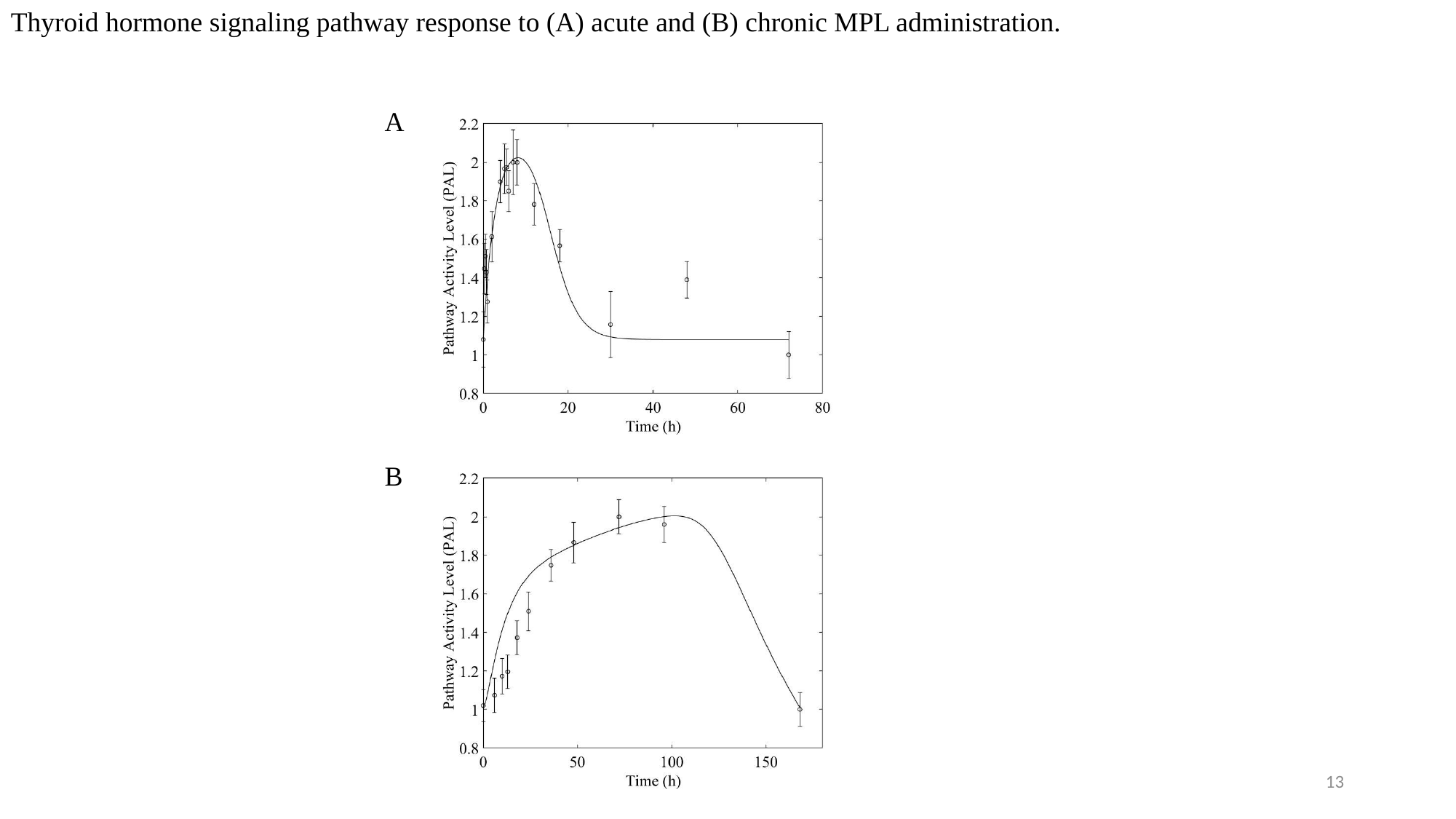

Thyroid hormone signaling pathway response to (A) acute and (B) chronic MPL administration.
A
B
13

## Slide 14
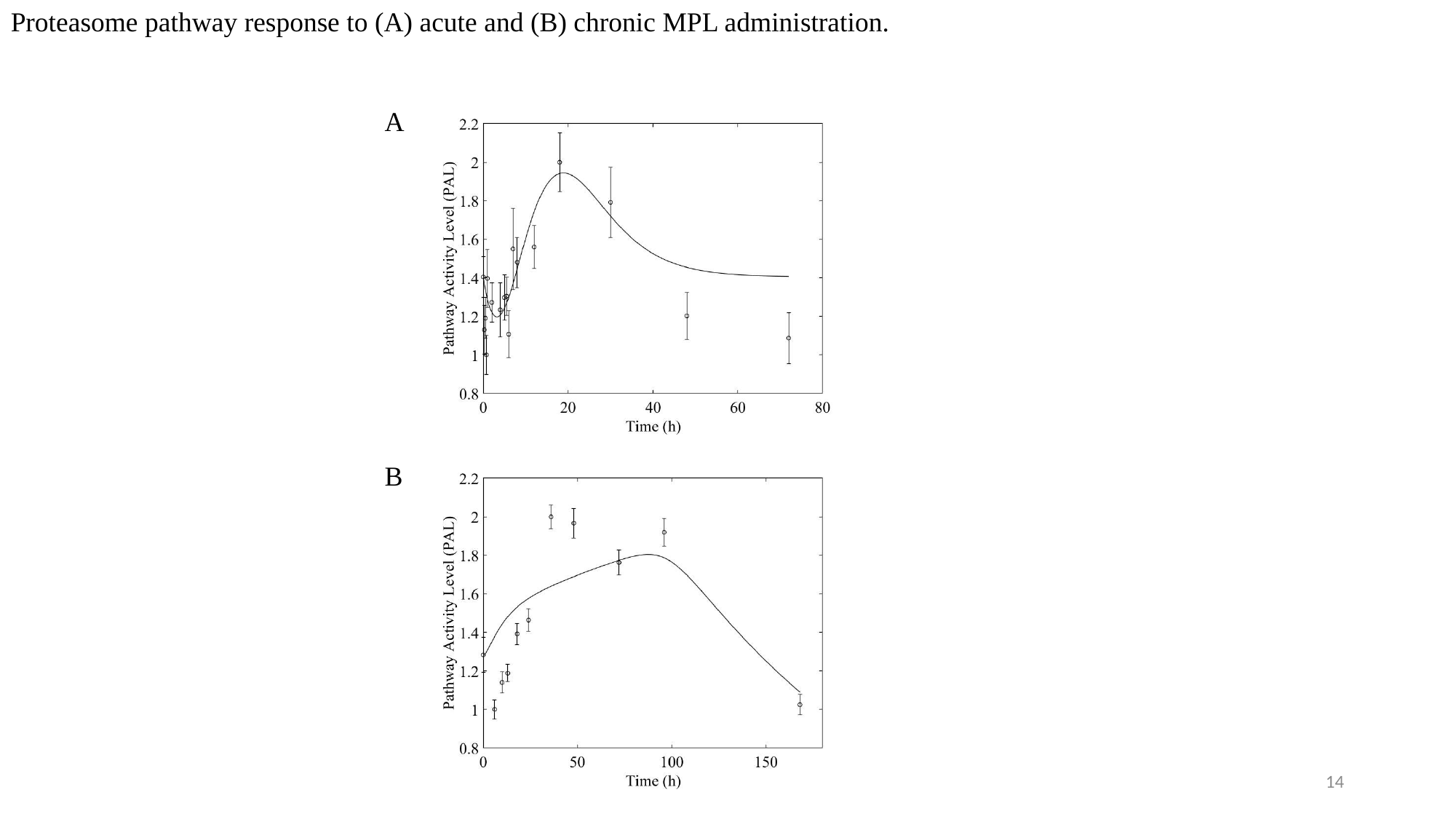

Proteasome pathway response to (A) acute and (B) chronic MPL administration.
A
B
14

## Slide 15
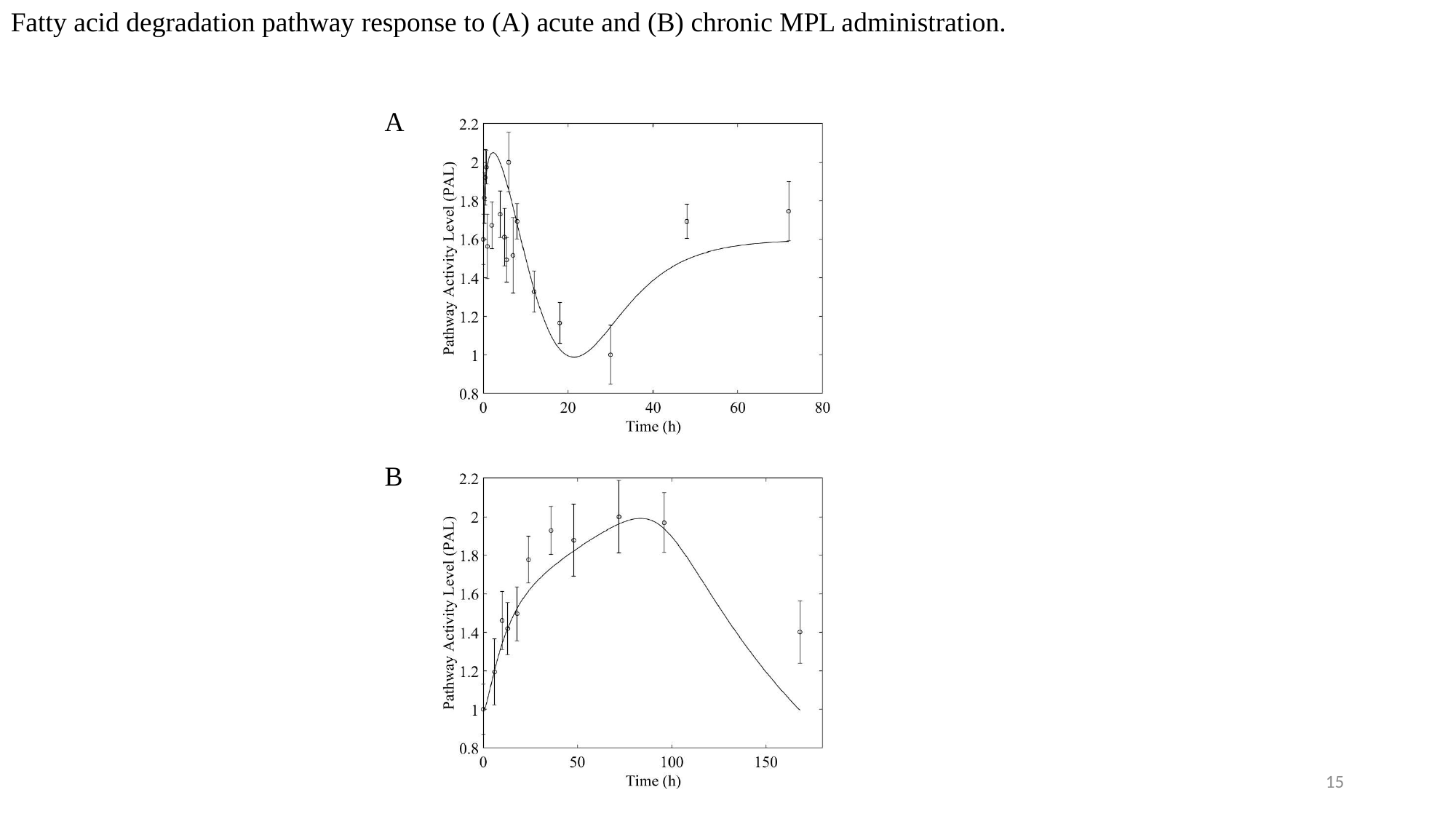

Fatty acid degradation pathway response to (A) acute and (B) chronic MPL administration.
A
B
15

## Slide 16
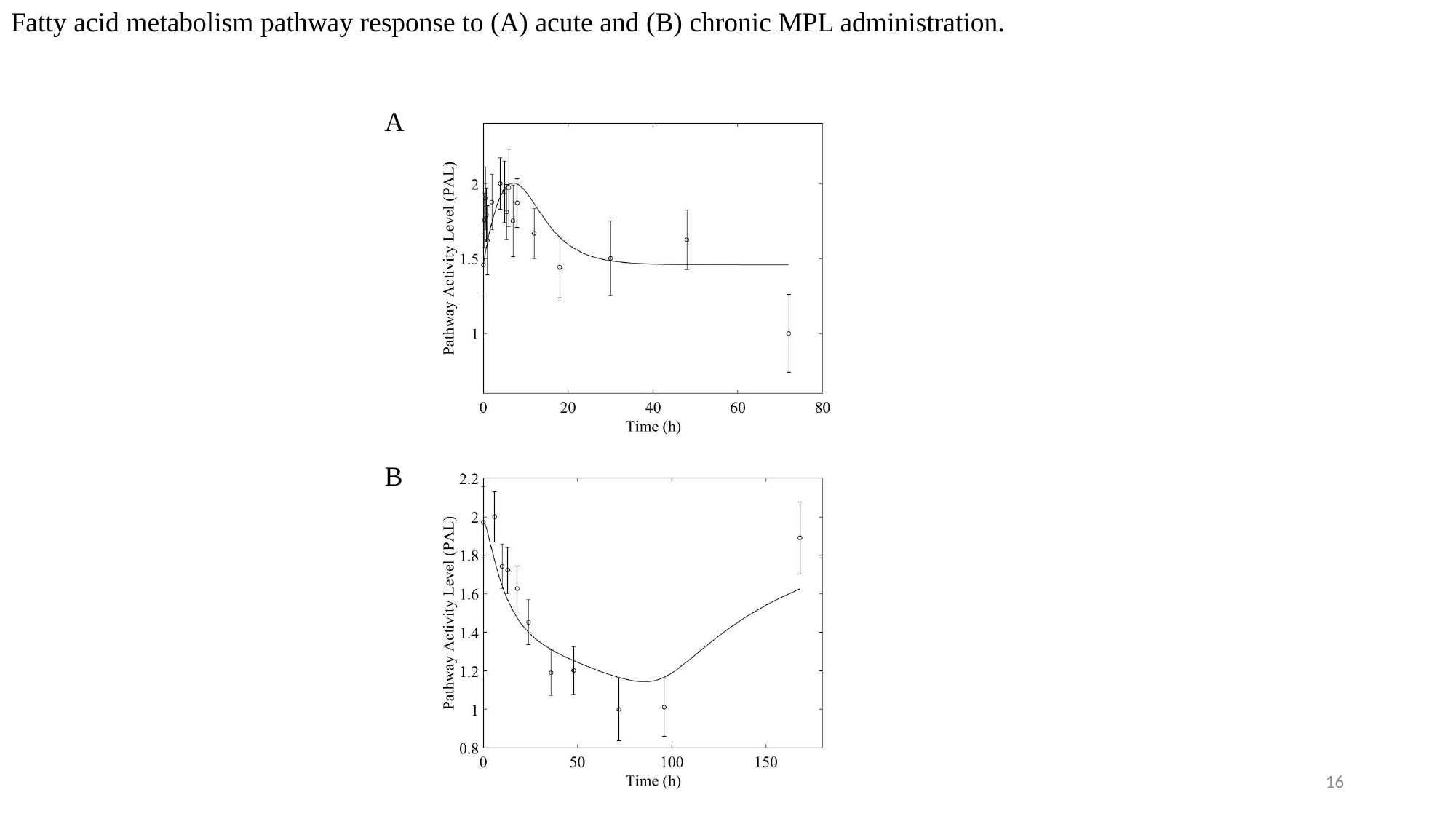

Fatty acid metabolism pathway response to (A) acute and (B) chronic MPL administration.
A
B
16

## Slide 17
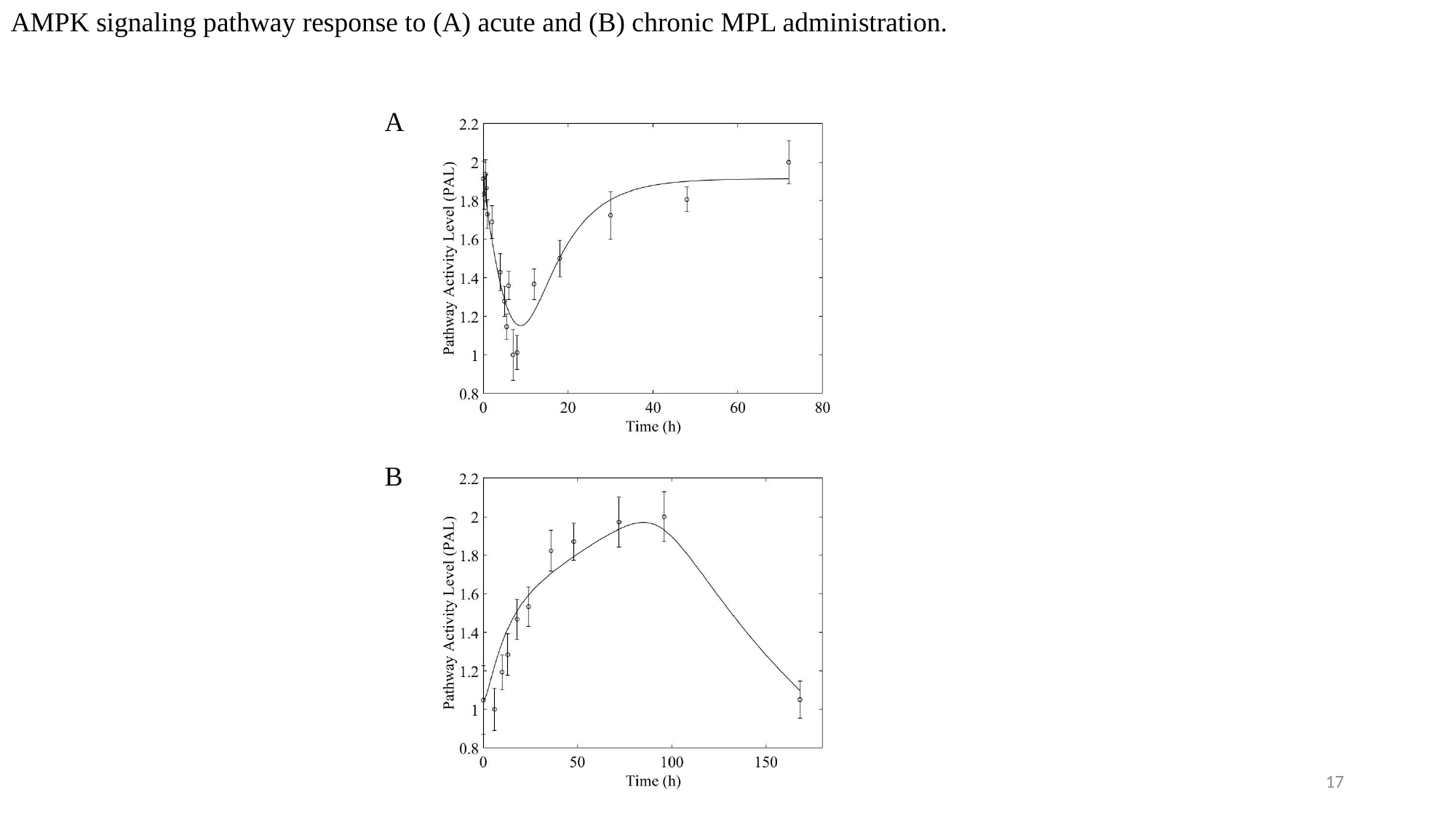

AMPK signaling pathway response to (A) acute and (B) chronic MPL administration.
A
B
17

## Slide 18
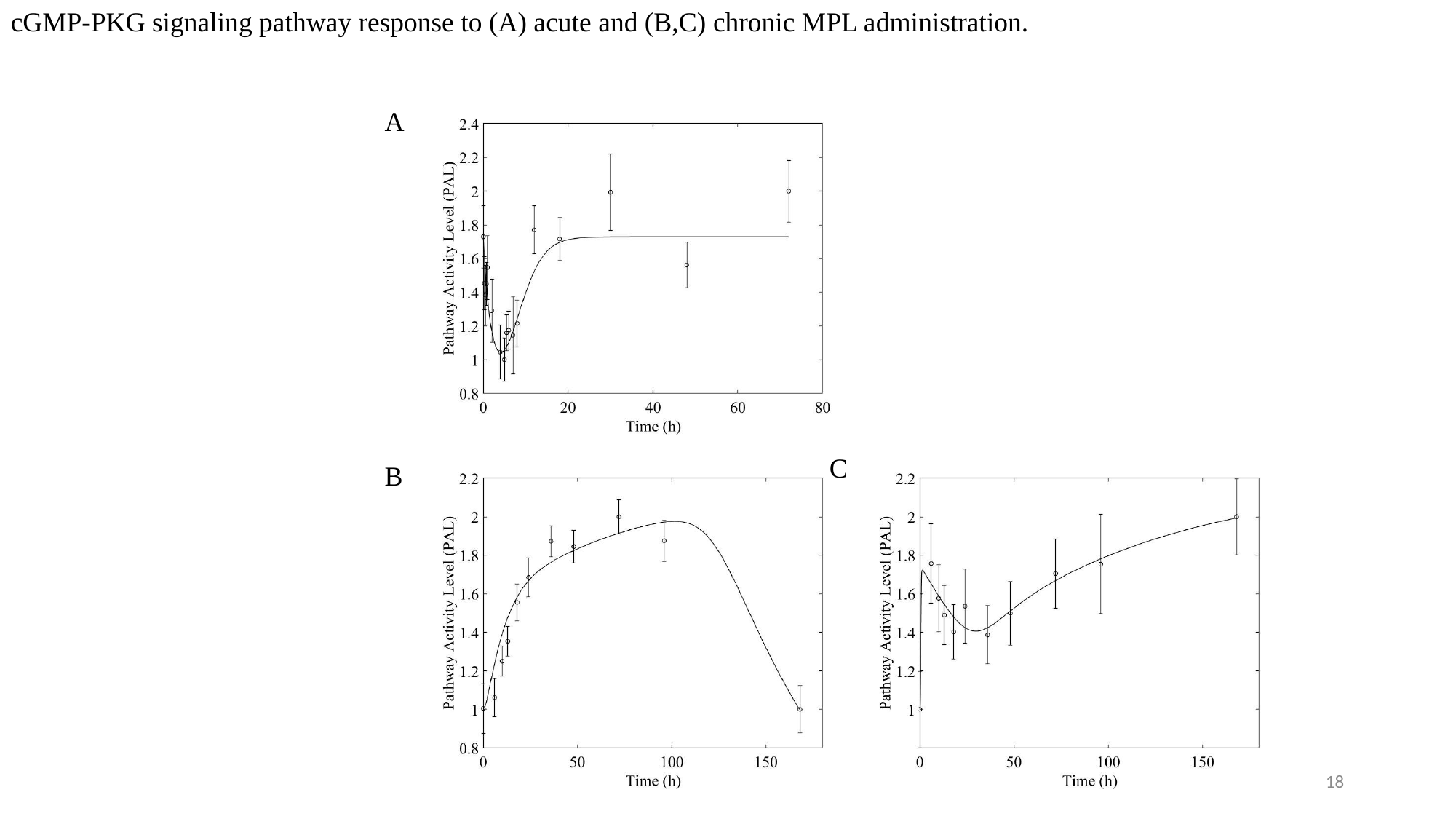

cGMP-PKG signaling pathway response to (A) acute and (B,C) chronic MPL administration.
A
B
C
18

## Slide 19
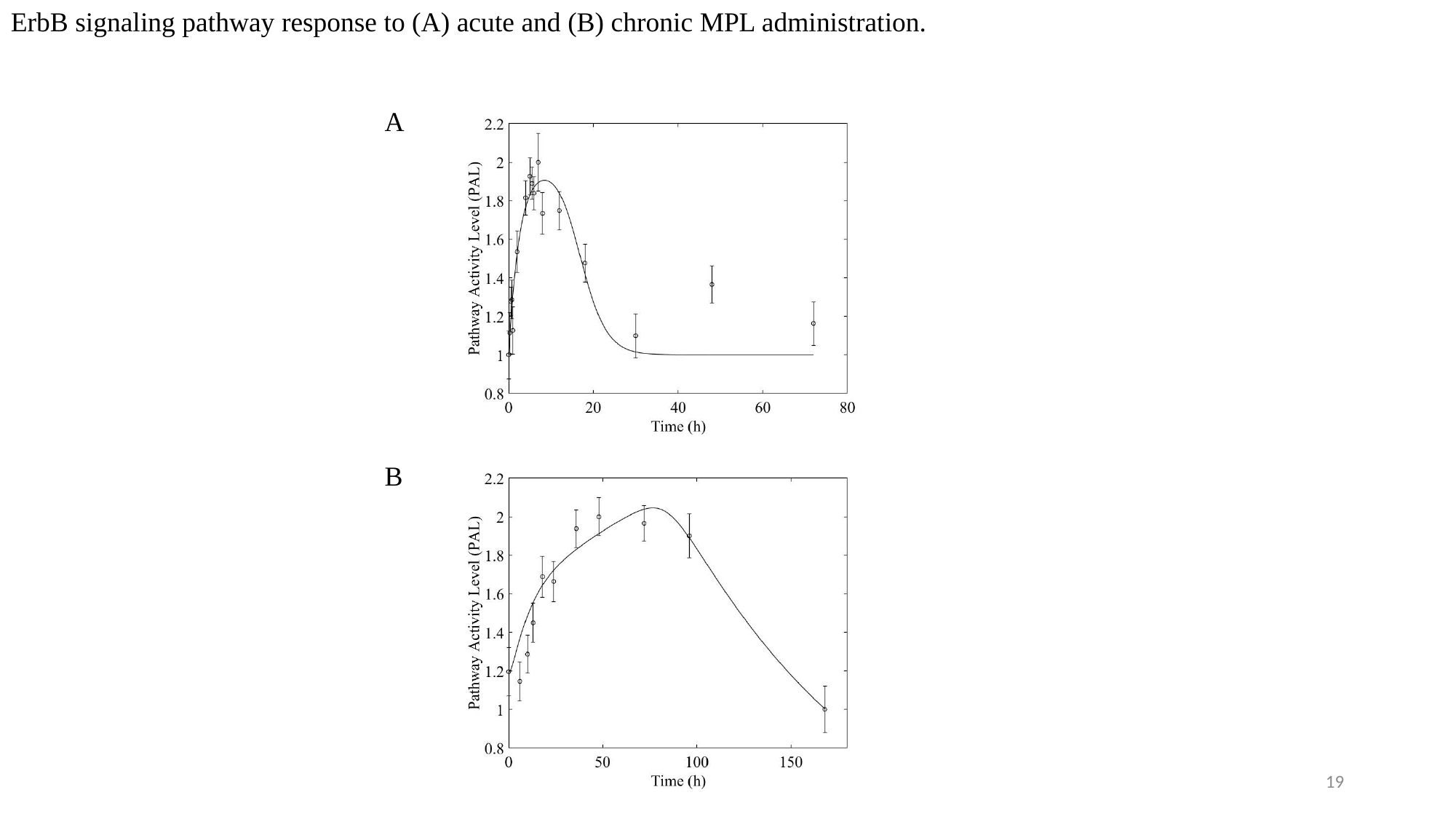

ErbB signaling pathway response to (A) acute and (B) chronic MPL administration.
A
B
19

## Slide 20
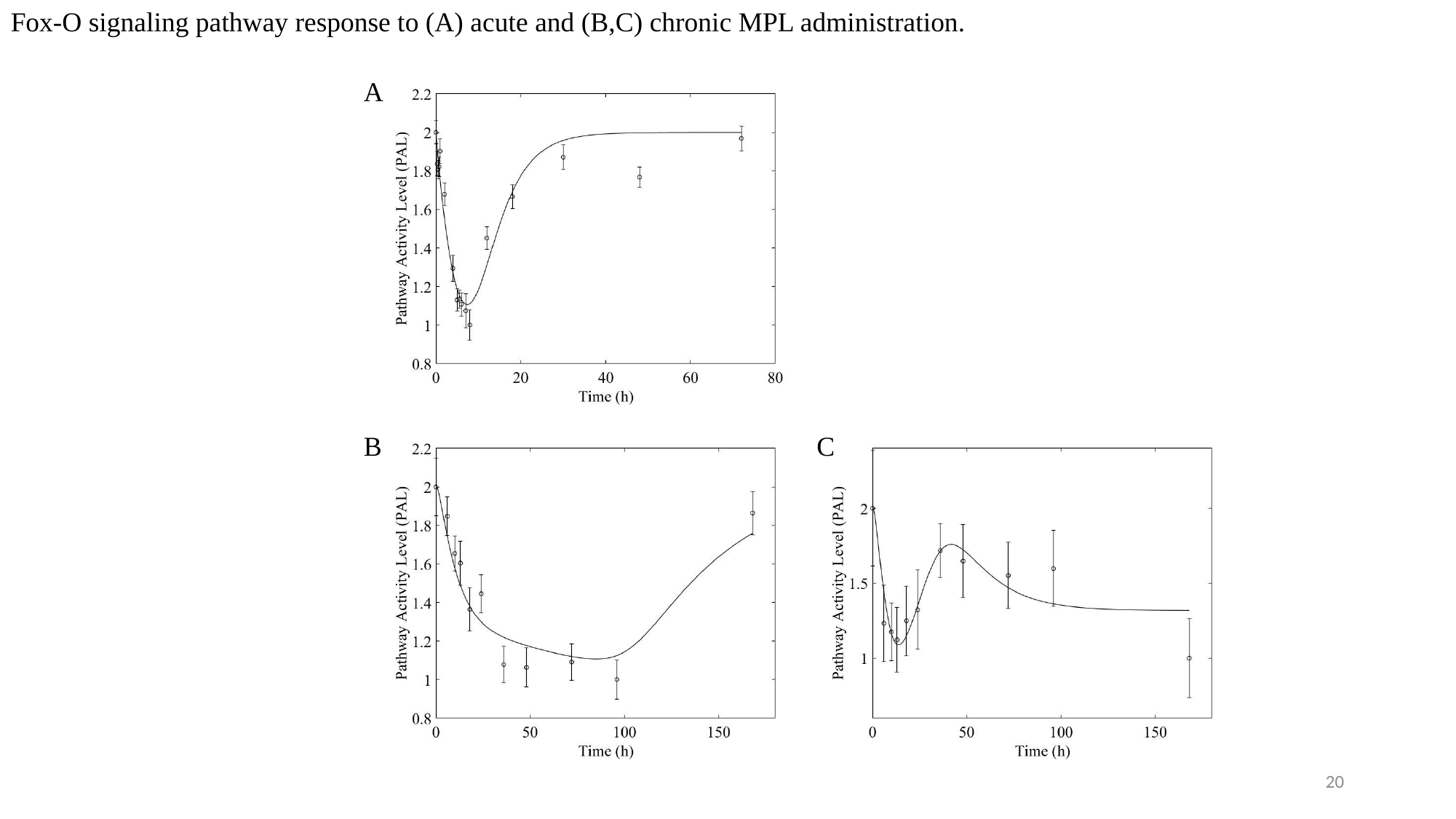

Fox-O signaling pathway response to (A) acute and (B,C) chronic MPL administration.
A
B
C
20

## Slide 21
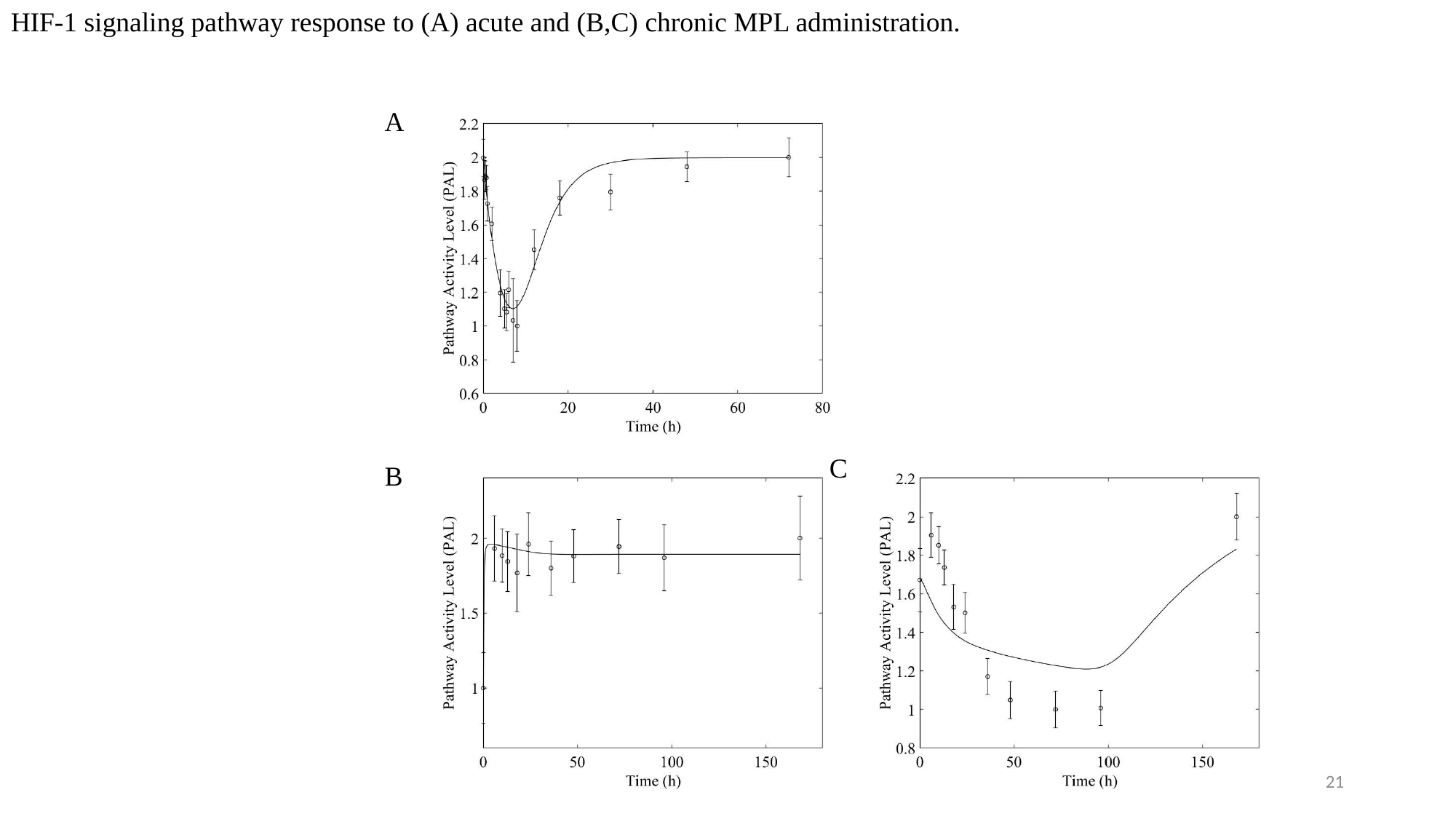

HIF-1 signaling pathway response to (A) acute and (B,C) chronic MPL administration.
A
B
C
21

## Slide 22
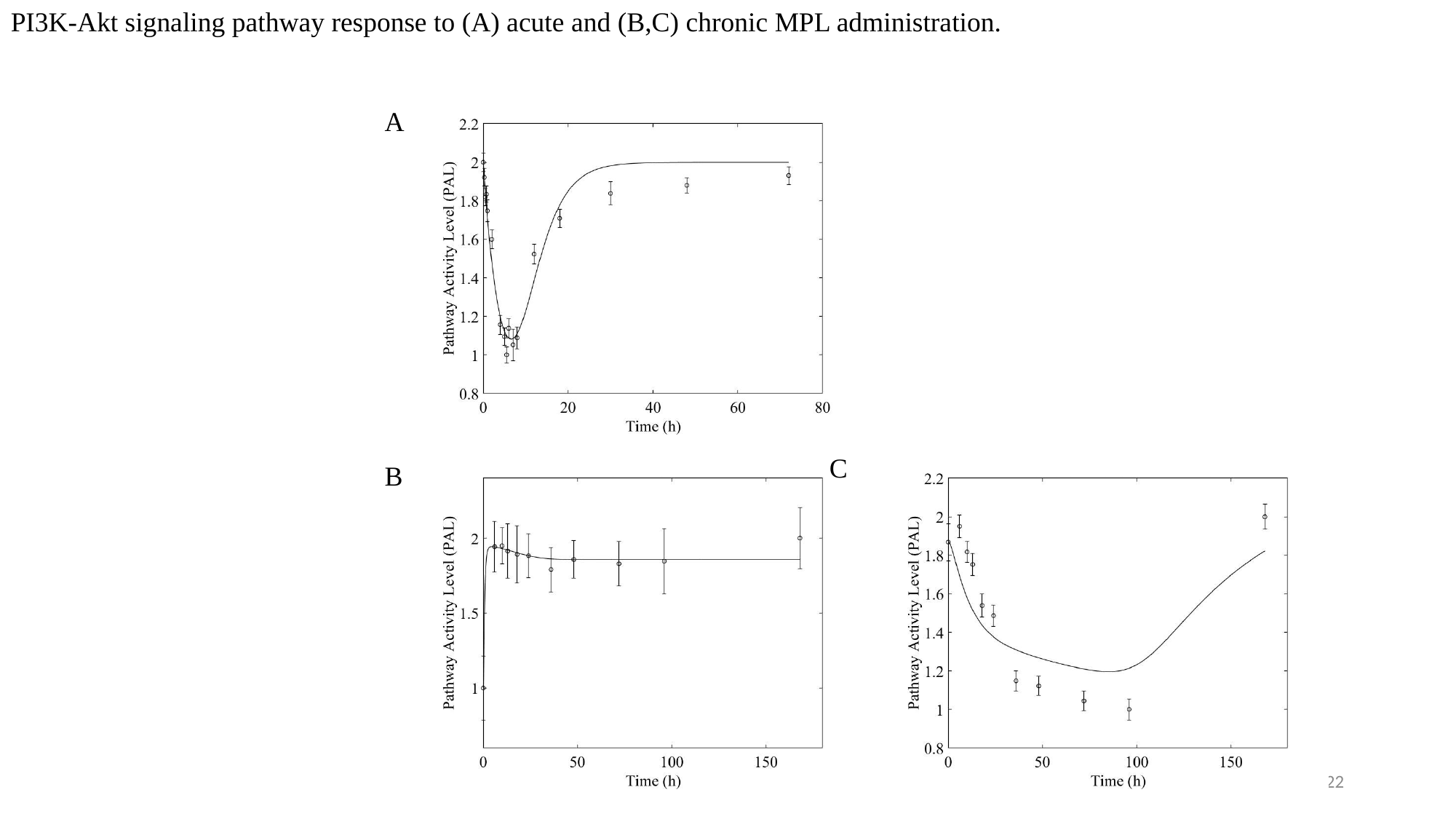

PI3K-Akt signaling pathway response to (A) acute and (B,C) chronic MPL administration.
A
B
C
22

## Slide 23
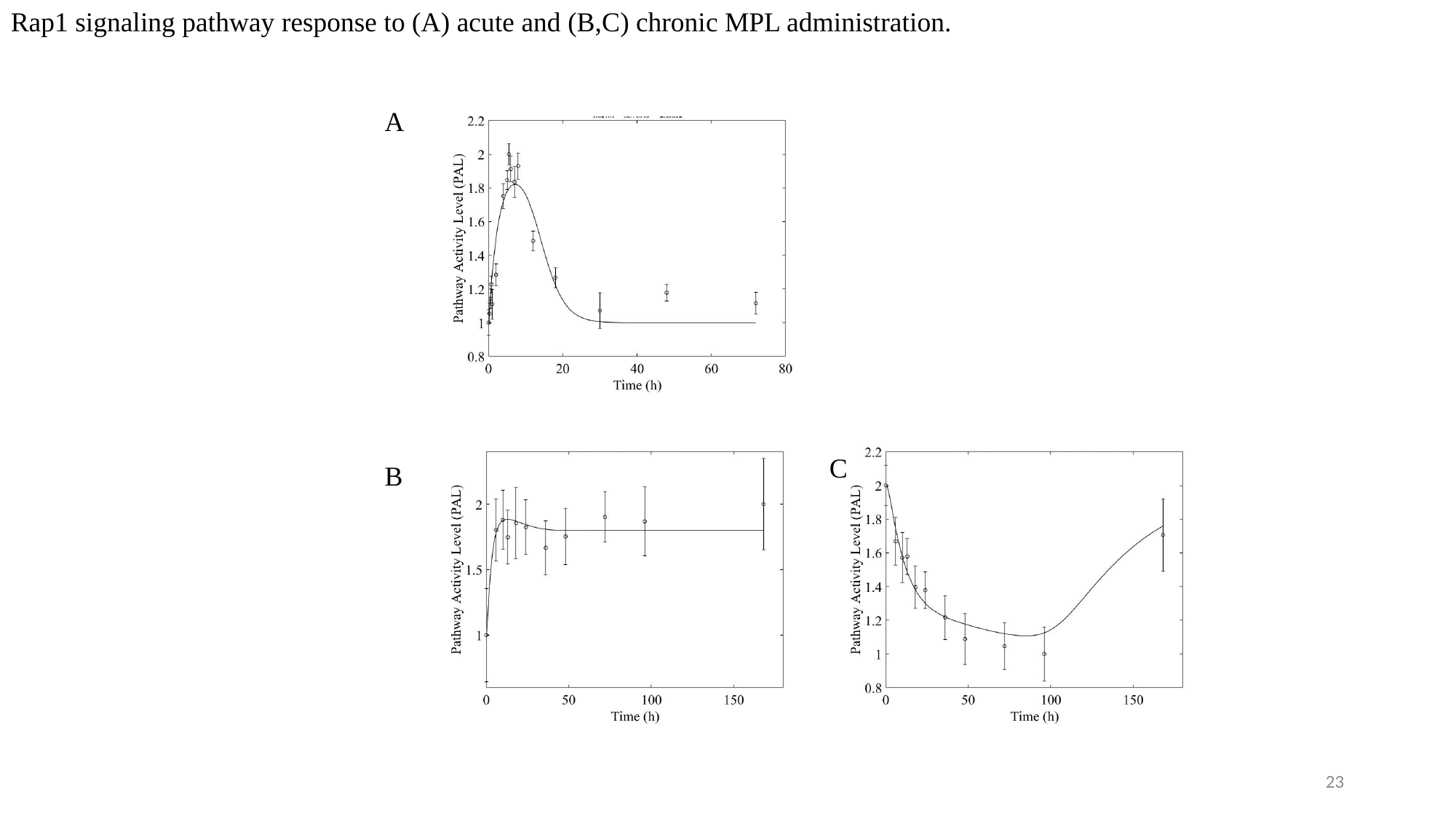

Rap1 signaling pathway response to (A) acute and (B,C) chronic MPL administration.
A
B
C
23

## Slide 24
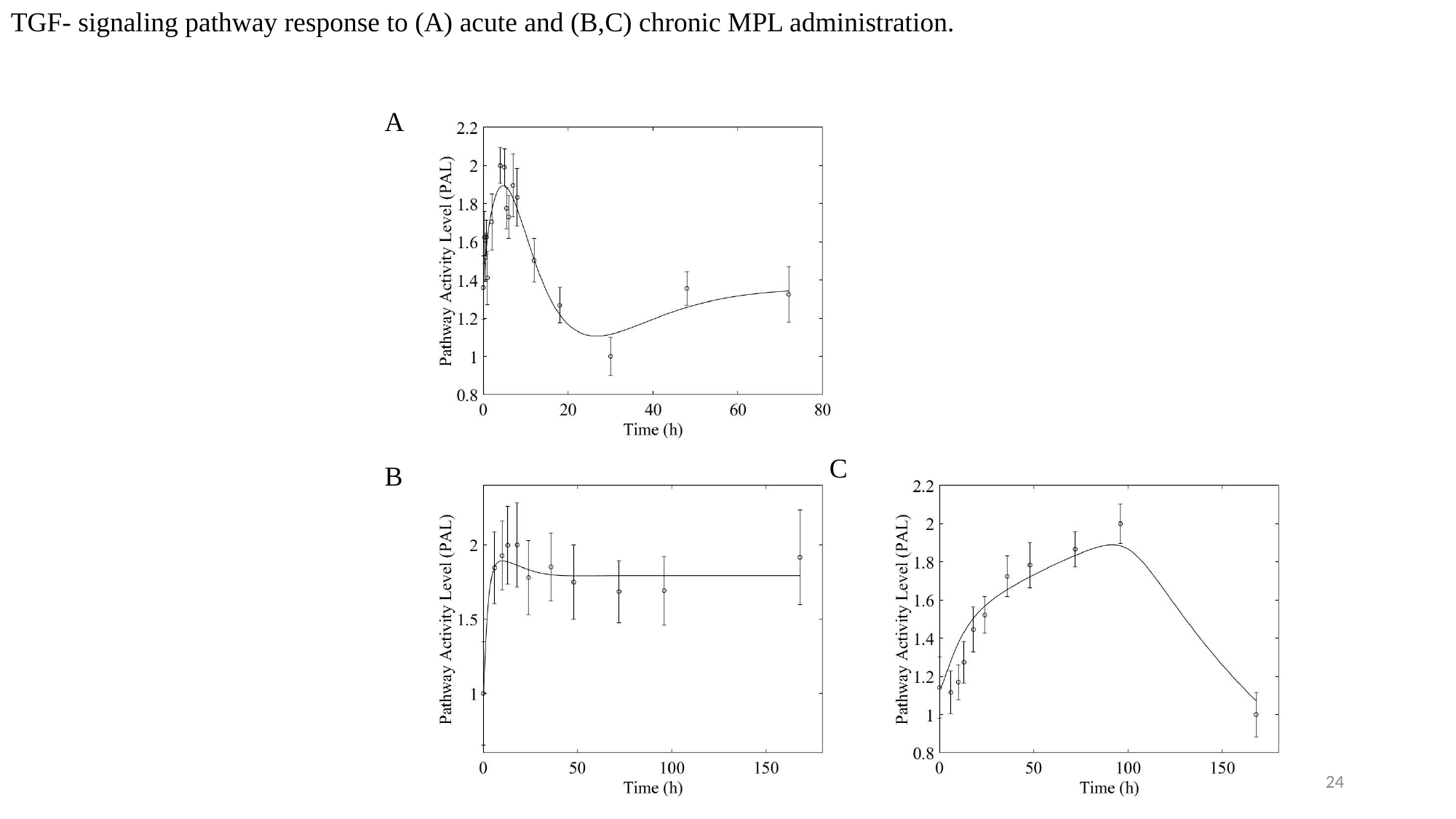

A
B
C
24

## Slide 25
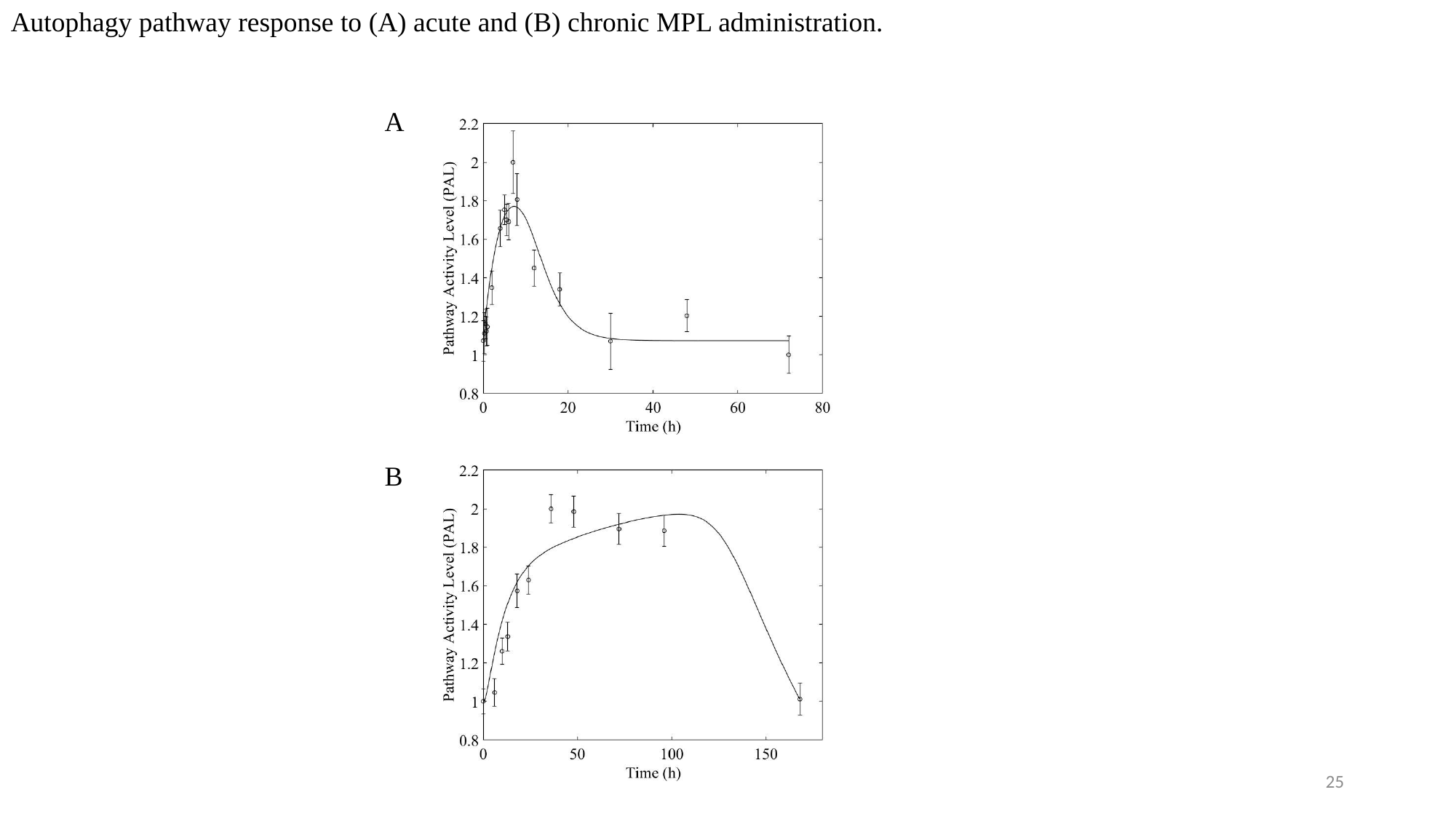

Autophagy pathway response to (A) acute and (B) chronic MPL administration.
A
B
25

## Slide 26
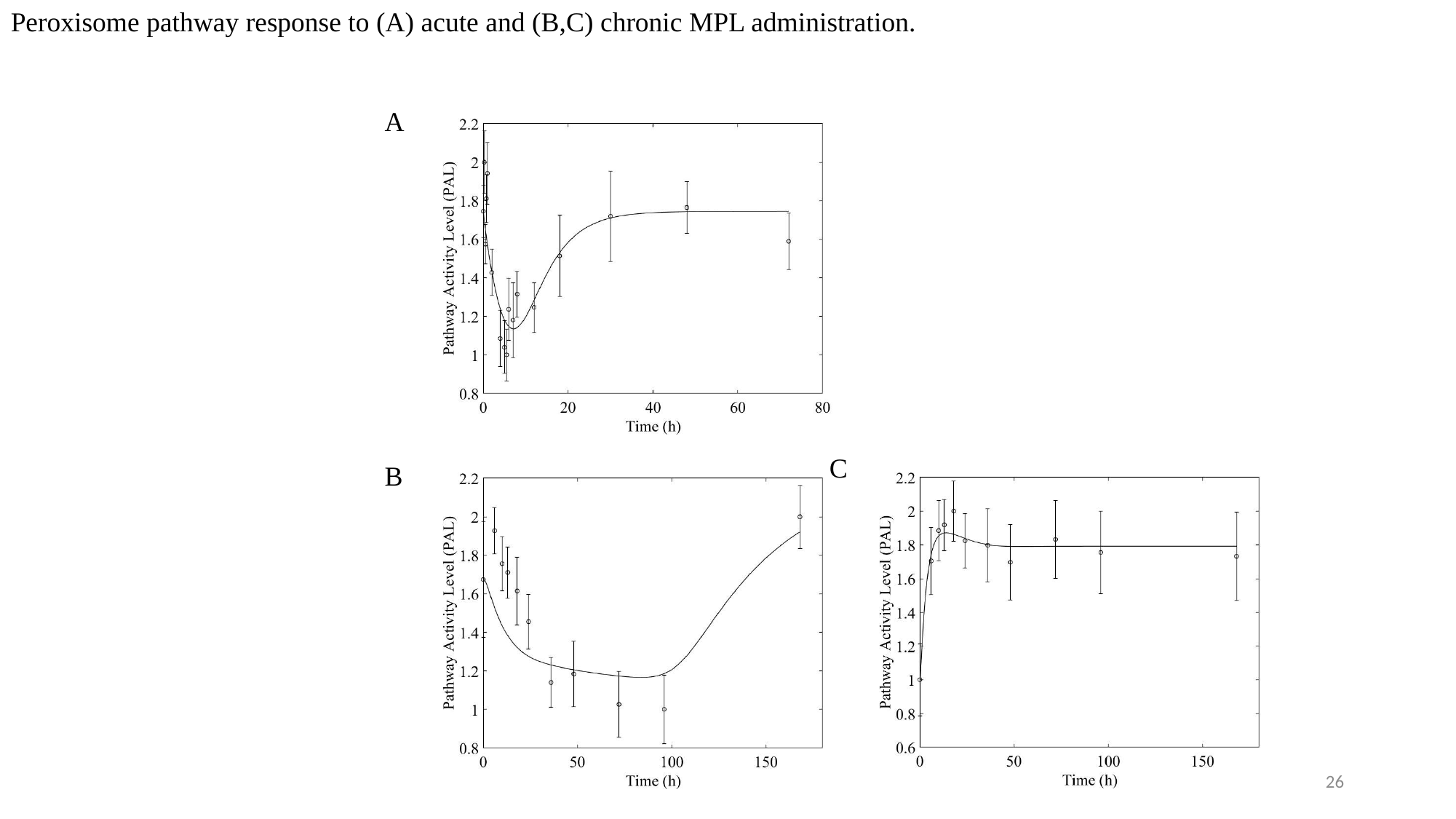

Peroxisome pathway response to (A) acute and (B,C) chronic MPL administration.
A
B
C
26

## Slide 27
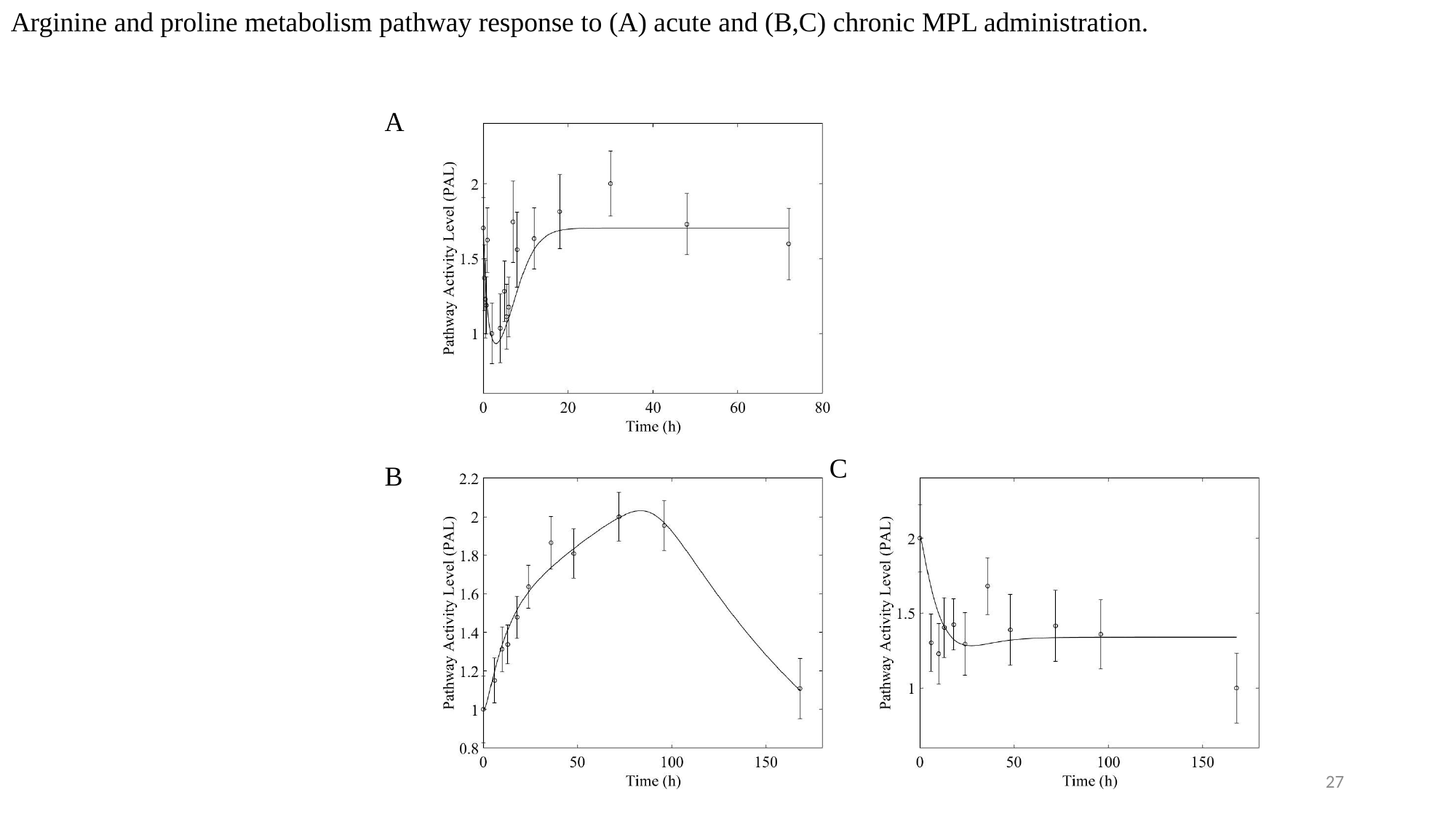

Arginine and proline metabolism pathway response to (A) acute and (B,C) chronic MPL administration.
A
B
C
27

## Slide 28
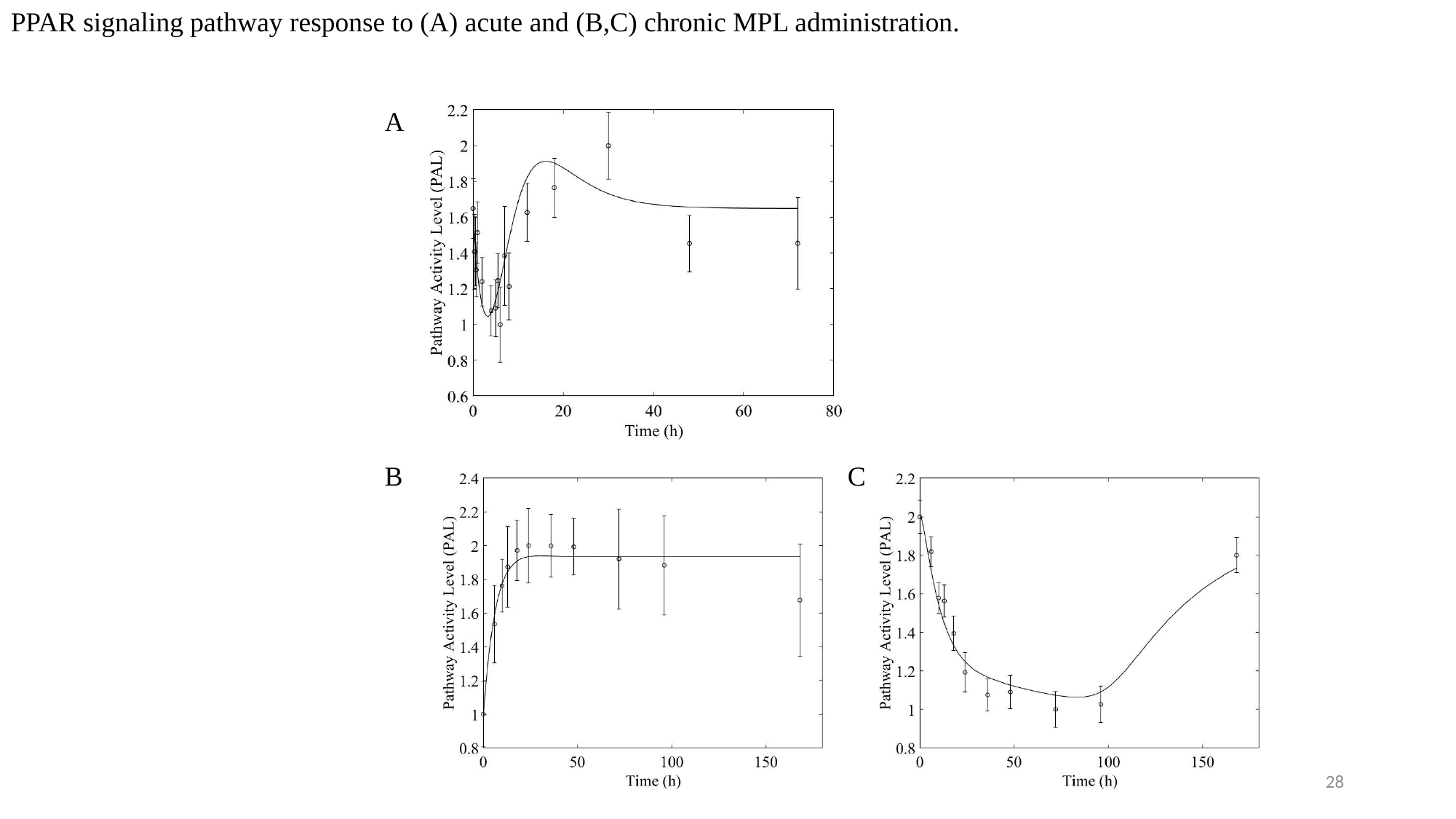

PPAR signaling pathway response to (A) acute and (B,C) chronic MPL administration.
A
B
C
28

## Slide 29
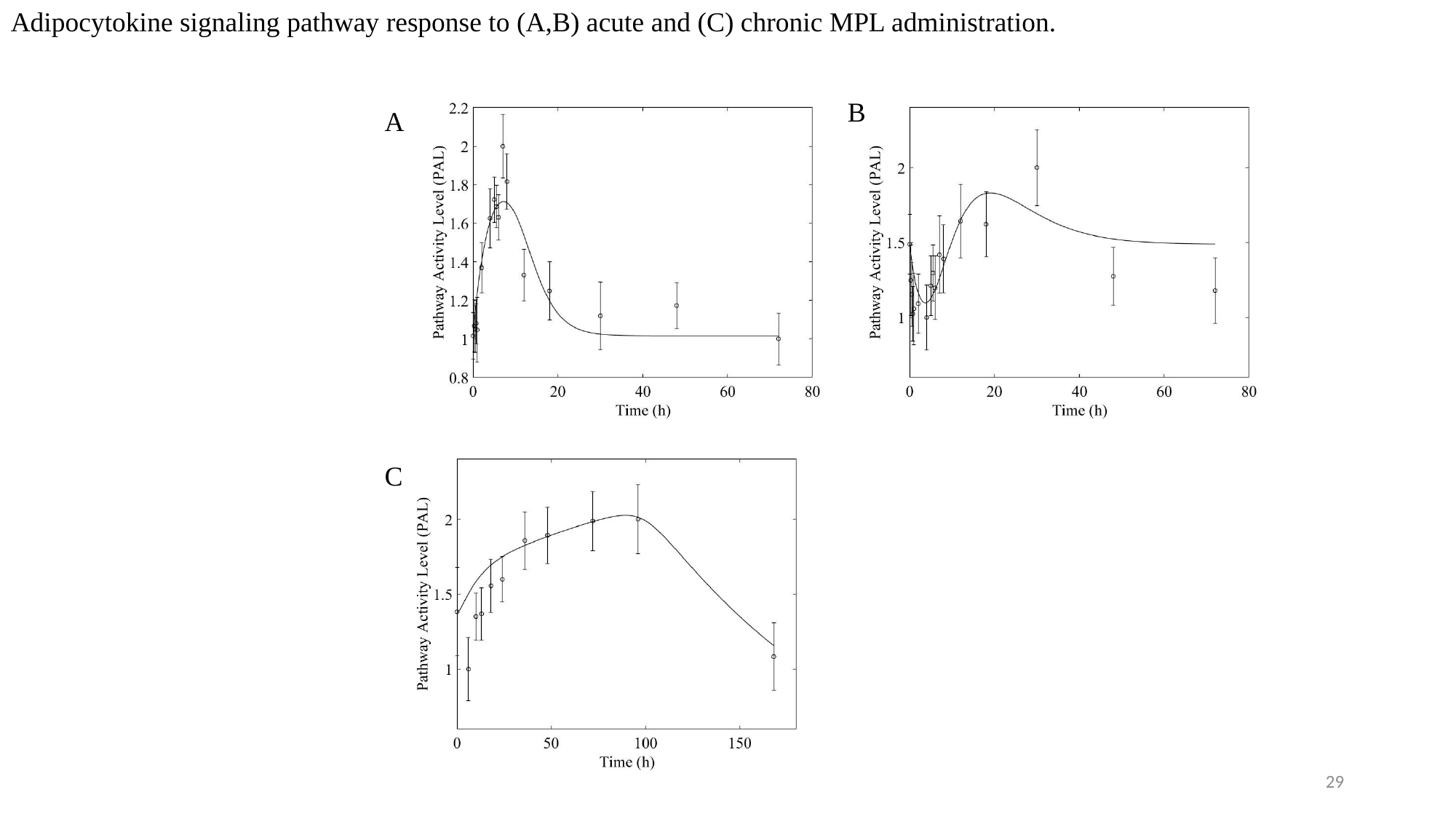

Adipocytokine signaling pathway response to (A,B) acute and (C) chronic MPL administration.
B
A
C
29

## Slide 30
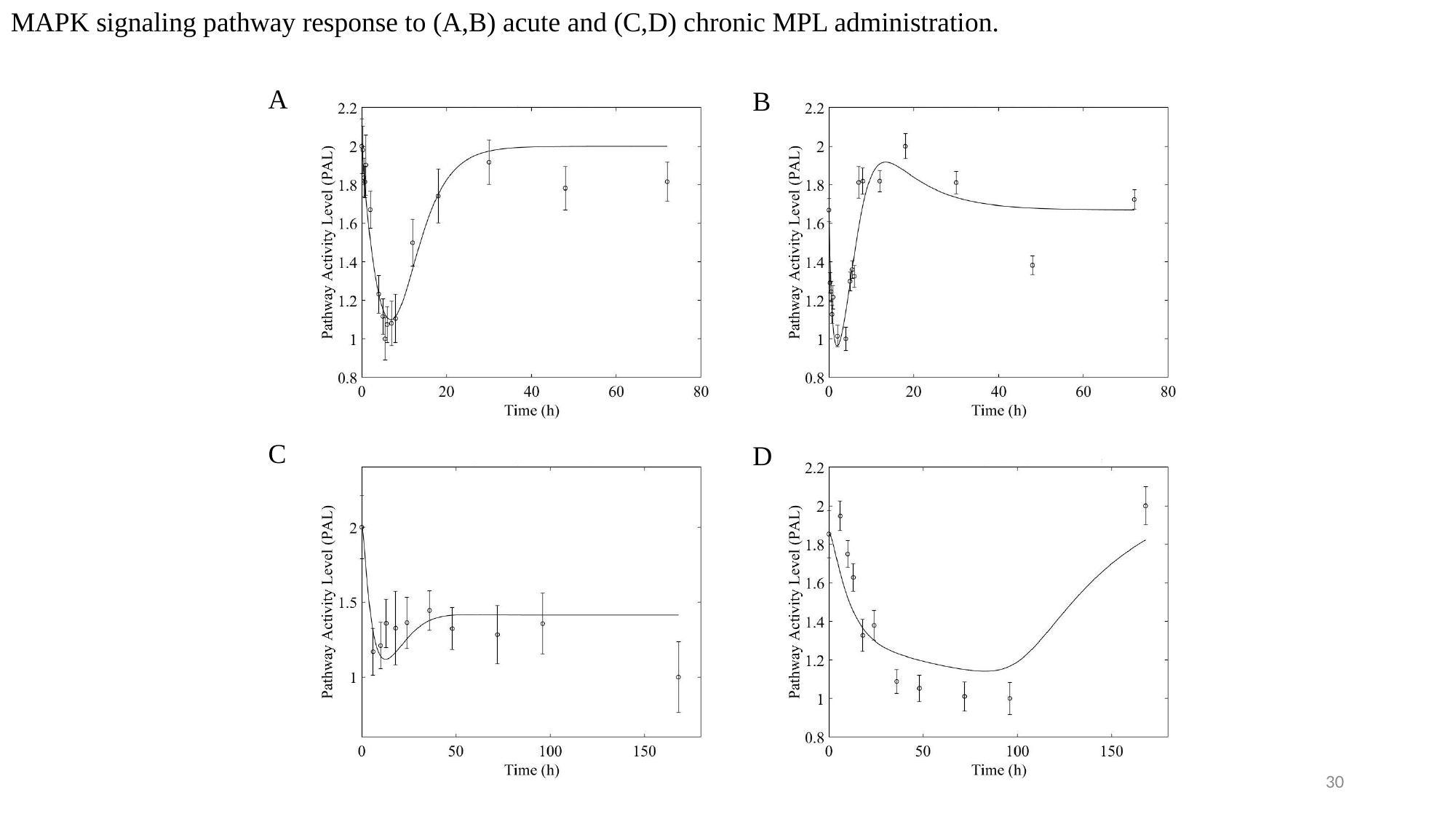

MAPK signaling pathway response to (A,B) acute and (C,D) chronic MPL administration.
A
C
B
D
30

## Slide 31
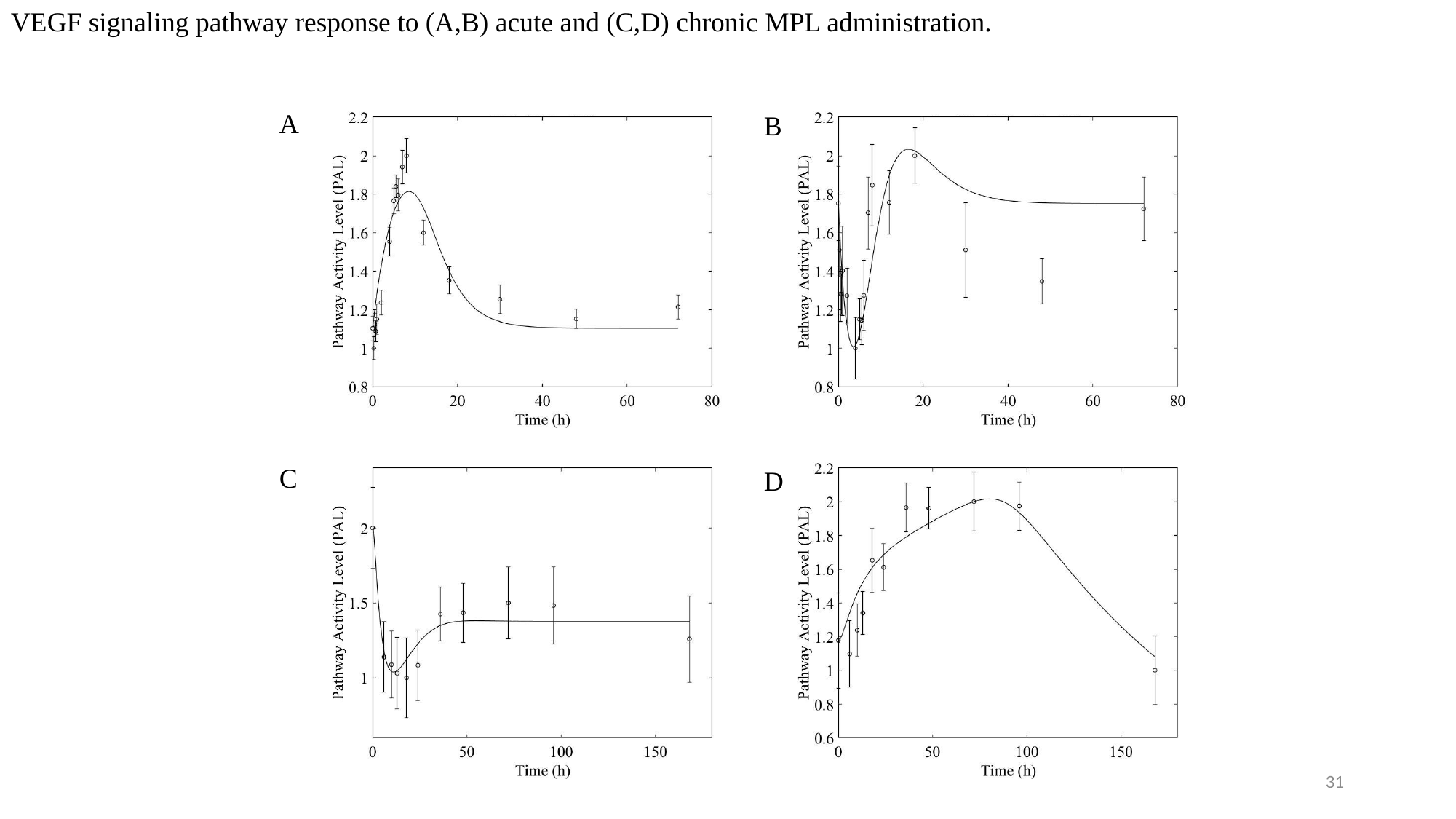

VEGF signaling pathway response to (A,B) acute and (C,D) chronic MPL administration.
A
C
B
D
31
