# Supplementary material for: Modeling Pathway Dynamics of the Skeletal Muscle Response to Intravenous Methylprednisolone (MPL) Administration in Rats: Dosing and Tissue Effects
Source: Front Bioeng Biotechnol. 2020 Jul 14;8:759. doi: 10.3389/fbioe.2020.00759 (PMC7371857; doi:10.3389/fbioe.2020.00759)
Supplement: Supplementary file 3 [file Table_2.docx]

**Supplementary Note**

**Calculating fp p-values using bootstrapping**

To determine PAL significance, a bootstrapping calculation is used to generate a p-value associated with $f_{p}$ statistic. The original gene set is bootstrapped ($N=1000$). Bootstrapped gene sets are constructed by scrambling the pathway gene set N times (Kallio, Vuokko et al. 2011). Each bootstrapped pathway gene set is decomposed with SVD, yielding $N$ sets of $PAL'$ profiles and associated $f_{p}'$ values for each $PAL'$ profile. For each PAL, the distribution of $f_{p}^{'}$ values which results from the bootstrapped pathway gene sets are compared to the original $f_{p}$ values. The number of $f_{p}^{'}$ greater than an $f_{p}$ is divided by N to determine whether each $f_{p}$ (and by association the PAL) is likely to emerge from a randomized gene set (Supplementary Note equation 1).

$f_{p} p-value=\frac{n_{\left( f_{p}^{'}>f_{p} \right)}}{N}$ Supplementary Note Eq. 1

**Calculating Significant PAL using Bootstrapping**

Variability exists in expression data capturing the influence of MPL within muscle endogenous expression in rat tissues, indicating non-uniformity that must be accounted for. To account for the variability, a bootstrapping approach is used to generate pathway gene sets likely to exist if this MPL dosing study were repeated, which are then assessed for pathway activity. In this component of the investigation, the range of activity capable of emerging from the system is investigated.

Bootstrapped gene sets are constructed from bootstrapped gene expression profiles, where each profile is projected within a normal distribution about the gene’s average expression. In short, each gene expression profile is bootstrapped within a normal distribution about the gene expression profile’s mean. The bootstrapped genes are assembled into appropriate pathway gene sets, ultimately yielding $N$ bootstrapped pathway gene sets for each pathway ($N=1000$ bootstrapped gene sets per pathway). Each of these bootstrapped pathway gene sets is decomposed with SVD. Significant PAL profiles identified from these bootstrapped gene and their corresponding $f_{p}$ and $f_{p}$ p-value statistics, are retained for each significant pathway. All PAL profiles extracted from these bootstrapped gene sets are assumed likely system behavior that would emerge if the rat experiments were repeated.

For each pathway, the significant bootstrapped PAL are clustered such that common activity patterns group together. The MATLAB ® function *evalclusters.m* is applied to assess optimal cluster number using the gap statistic and applying kmeans clustering (MATLAB 2016b). Thus, a finite set of PAL centroids are identified, indicating a finite list of activity patterns emerge from each pathway in response to MPL. The figures within the main text that report PAL profiles are plots of the centroids of these clusters (represented by the data points), fitted with models (represented by the fitted continuous profile). The error bars about the PAL central data points are defined by the standard deviation of all bootstrapped PAL that are captured within the cluster.
